# Gender-related differences in prevalence, intensity and associated risk factors of *Schistosoma* infections in Africa: A systematic review and meta-analysis

**DOI:** 10.1371/journal.pntd.0009083

**Published:** 2021-11-17

**Authors:** Diepreye Victoria Ayabina, Jessica Clark, Helena Bayley, Poppy H. L. Lamberton, Jaspreet Toor, T. Deirdre Hollingsworth

**Affiliations:** 1 Big Data Institute, Li Ka Shing Centre for Health Information and Discovery, University of Oxford, Oxford, United Kingdom; 2 Institute of Biodiversity, Animal Health and Comparative Medicine, University of Glasgow, Glasgow, United Kingdom; 3 Wellcome Centre for Integrative Parasitology, University of Glasgow, Glasgow, United Kingdom; 4 Department of Physics, University of Oxford, Oxford, United Kingdom; 5 MRC Centre for Global Infectious Disease Analysis, School of Public Health, Imperial College London, London, United Kingdom; Universidade Federal de Alagoas - Campus Arapiraca, BRAZIL

## Abstract

**Background:**

Schistosomiasis remains a global-health problem with over 90% of its burden concentrated in Africa. Field studies reflect the complex ways in which socio-cultural and socio-economic variables, affect the distribution of *Schistosoma* infections across different populations. This review set out to systematically investigate and quantify the differences in *Schistosoma* infection burdens between males and females in Africa for two of the most prevalent *Schistosoma* species—*Schistosoma mansoni* and *Schistosoma haematobium*.

**Methodology:**

We searched (from inception to 11th March 2020) Embase, MEDLINE, PubMed, and Web of Science for relevant studies on schistosomiasis. We included studies that report *S*. *mansoni* and/or *S*. *haematobium* prevalence and/or intensity data distributed between males and females. We conducted meta-analyses on the male to female (M:F) prevalence of infection ratios. Subgroup analyses were performed according to study baseline prevalence, sample size and the lower and upper age limit of study participants. We also present a descriptive analysis of differential risk and intensity of infection across males and females. Evidence for differences in the prevalence of schistosomiasis infection between males and females is presented, stratified by *Schistosoma* species.

**Result:**

We identified 128 relevant studies, with over 200,000 participants across 23 countries. Of all the reported differences in the prevalence of infection between males and females, only 41% and 34% were statistically significant for *S*. *mansoni* and *S*. *haematobium*, respectively. Similar proportions of studies (27% and 34% for for *S*. *haematobium* and *S*. *mansoni*, respectively) of the reported differences in intensity of infection between males and females were statistically significant. The meta-analyses summarized a higher prevalence of infection in males; pooled random-effects weighted M:F prevalence of infection ratios were 1.20 (95% CI 1.11–1.29) for *S*. *haematobium* and 1.15 (95% CI 1.08–1.22) for *S*. *mansoni*. However, females are underrespresented in some of the studies. Additionally, there was significant heterogeneity across studies (Higgins *I*^2^ statistic (p-values < 0.001, *I*^2^*values*>95%)). Results of the subgroup analysis showed that the baseline prevalence influenced the *M*:*F* prevalence ratios for *S*. *haematobium* and *S*. *mansoni*, with higher M:F prevalence of infection ratios in settings with a lower baseline prevalence of infection. Across the studies, we identified four major risk factors associated with infection rates: occupational and recreational water contact, knowledge, socio-economic factors and demographic factors. The effect of these risk factors on the burden of infection in males and females varied across studies.

**Conclusions:**

We find evidence of differences in prevalence of infection between males and females which may reflect differences in gender norms and water contact activities, suggesting that policy changes at the regional level may help ameliorate gender-related disparities in schistosomiasis infection burden. Collecting, robustly analysing, and reporting, sex-disaggregated epidemiological data, is currently lacking, but would be highly informative for planning effective treatment programmes and establishing those most at risk of schistosomiasis infections.

## Author summary

Schistosomiasis is a parasitic waterborne disease affecting more than 240 million people worldwide every year, especially in sub-Saharan Africa. The difference in *schistosoma* infection prevalence, infection intensity and risk between males and females has rarely been quantified. In this study, we aim to qualitatively and quantitatively synthesise published studies that present sex-disaggregated epidemiological data and investigate gender differences (e.g. those driven by social and cultural norms) that predispose males or females to higher risk of infection. Based on the 123 studies included in our meta-analysis, we found a higher prevalence of Schistosoma (*S*. *mansoni* and *S*. *haematobium*) in males.

## Introduction

Schistosomiasis is a neglected tropical disease (NTD) with over 240 million people infected. Over the last decade, progress has been made towards achieving schistosomiasis morbidity control in several countries in the sub-Saharan African region, although more remains to be done [1]. The World Health Organisation (WHO) strategy for schistosomiasis control focuses on reducing disease through periodic, targeted treatment with praziquantel through the large-scale treatment (preventive chemotherapy) of affected populations. About 92% of those requiring preventive treatment live in sub-Saharan Africa [2]. Generally, the highest prevalence and intensity of infections have been found in school-aged children (SAC; 5–14 years old) [3], though pre-SAC and adults can also be highly infected [4,5]. The disease is caused by parasitic flukes of the genus *Schistosoma* and manifests in two forms; urogenital schistosomiasis (*Schistosoma haematobium*) and intestinal schistosomiasis (*Schistosoma mansoni*, *Schistosoma japonicum*, *Schistosoma mekongi*, *Schistosoma guineensis* and *Schistosoma intercalatum*). Although the epidemiology of each species differs to a certain extent, transmission requires contamination of fresh water by faeces (intestinal) or urine (urogenital) containing eggs, an intermediate snail host, and human contact with that contaminated water inhabited by the intermediate host snail. Humans are infected by the cercarial aquatic larval stage of the parasite, with exposure depending on a range of factors which could be based on individual, socio-economic or socio-cultural behaviours. The two major *Schistosoma* species prevalent in sub-Saharan Africa are *S*. *mansoni* and *S*. *haematobium* which are transmitted via intermediate snail hosts *Biomphalaria sp*. and *Bulinus sp*., respectively [6].

Host gender shapes peoples risk of *schistosoma* infections [7]. Local differences in prevalence and intensity observed between men and women may, in part, be explained by differences in the social and occupational roles taken up by men and women affecting water-related contact, including frequency, duration and level of submergence, which can all be influenced by age and cultural beliefs [8]. For example, in many African countries, activities including washing clothes, bathing children and rice cultivation are mostly carried out by women and girls, thus increasing their risk of schistosomiasis [9]. On the other hand, previous work has suggested that males typically suffer from a higher parasitic infection risk than females [10] and based on studies in Puerto Rico and Brazil, fishing and swimming are more often associated with males [9]. Additionally, as children mature, the frequency and duration of water contact can change, dependent on age and gender, resulting in divergent infection dynamics [11]. Despite these general observations, the difference in *schistosoma* infection prevalence, infection intensity and risk between males and females has rarely been quantified [8]. A review by Sevilimedu et al [12], on the gender distribution of diarrheal disease-causing pathogens showed higher prevalence of *S*. *mansoni* in males. This study was geographically broad however it did not include *S*. *haematobium*. Given the prevalence of infection in Africa and that the *Schistosoma* species most prevalent in Africa are *S*. *mansoni* and *S*. *haematobium*, there is a need to provide updated findings specifically for Africa, whilst also expanding these results to include *S*. *haematobium* as well as *S*. *mansoni*.

The aim of this study is to investigate the differences in prevalence and intensity of *S*. *mansoni* and *S*. *haematobium* infections between males and females in Africa. The question which we aim to answer is: in individuals that have *Schistosoma* infections (*S*. *haematobium* or *S*. *mansoni*), do males or females have significantly higher prevalence and/or intensity of infection? We aim to qualitatively and quantitatively synthesise published studies that present sex-disaggregated epidemiological data and investigate gender differences (e.g. those driven by social and cultural norms) that predispose males or females to higher risk of infection. We note that, whilst the burden of schistosomiasis can be considered in many forms (e.g. infection prevalence, intensity, morbidity, or socio-economic), and whilst none of these are mutually exclusive, investigating gender-related differences in all of these simultaneously, is outwith the scope of this paper. Understanding the distribution of *Schistosoma* infection prevalence and intensity between males and females can support programmes to understand and identify who is most infected with *S*. *mansoni* and *S*. *haematobium*, contributing to transmission and subsequent morbidity.

## Methods

This systematic review is reported in accordance with the Preferred Reporting Items for Systematic Reviews and Meta-Analyses (PRISMA) guidelines, the completed checklist for which is in S1 Table. A protocol was prospectively registered in PROSPERO, registration number: CRD42020175165.

### Search strategy

The lead author, DA, searched the following databases (from inception to 31st March 2020): Embase, MEDLINE, PubMed, and Web of Science. The search terms used were derived from two general sections: the disease and the epidemiological measures of interest. Variations on the following keywords were used for the search “schistosomiasis”, “prevalence” and “intensity”. We used Boolean operators “AND” to combine the categories and “OR” to join the terms within each category. Exact search terms are provided in S1 Text. To not limit the span of the search we did not include search terms relating to Africa, or specific countries within Africa. Based on expert recommendation, we also manually searched the Schistosomiasis Consortium for Operational Research and Evaluation (SCORE) for any additional papers not picked by our search strategy. Identified studies were imported into EndNote X9 (Thomson Reuters, New York, USA) and duplicates were removed.

### Selection criteria

The remaining unique studies were reviewed by titles, abstracts, figures and tables by DA, JC and JT independently and in duplicate. In case of any disagreement, a final decision was reached by team consensus. Following the participants, intervention, comparison, outcomes, study (PICOS) design criteria, we included studies assessing the following:

- Population: Human population (no age or geographical restriction) diagnosed with *Schistosoma* infections (*S*. *haematobium* or *S*. *mansoni*).
- Intervention or exposure: Exposure to *S*. *haematobium* or *S*. *mansoni*.
- Comparison: The comparison will be *Schistosoma* infection prevalence and/or intensity between male and female participants.
- Outcomes: The outcome measures are the male to female ratios of prevalence and/or intensity of infection.
- Study design: Observational studies.

We included observational studies written in English (for details on any publication bias, see S2 Text and S1 and S2 Figs), that reported the prevalence and/or intensity of *Schistosoma* infections (*S*. *mansoni* and/or *S*. *haematobium*) in males and females in Africa. We excluded studies that (i) did not report prevalence and/or intensity separately for males and females (or men and women, boys and girls), (ii) full publication could not be obtained, (iii) extended analysis of previously published studies, (iv) previously published reviews on the epidemiology of schistosomiasis as these papers did not contain any new data, (v) non-human studies, and (vi) studies on human schistosomiasis caused by other *Schistosoma* species. Unpublished manuscripts, conference proceedings, conference abstracts, editorials, commentaries, letters to editors and author replies were also excluded.

### Data extraction

Relevant data were extracted from eligible publications into a predefined Microsoft Excel extraction sheet developed for this review. For each full text included, the following information was extracted:

1. Publication details: title, journal, author(s), year of publication, country and year in which study was carried out
2. Study design: type of study, sampling method, *Schistosoma* species, diagnostic method(s)
3. Study population demographics (participants): number of individuals in study, population characteristics including age and demographic information.
4. Outcome measures: the main outcome variables extracted were male and female prevalence, and mean intensity of infection (defined as the mean number of schistosome eggs per gram of stool (for *S*. *mansoni*) and eggs per 10 millilitres of urine (for *S*. *haematobium*) in a population). We use the prevalence of infection and intensity of infection in males and females to calculate the male to female (*M*:*F*) prevalence and intensity of infection ratios. We did not differentiate between studies which presented results based on host sex (male/female) or gender (man/woman, boy/girl) and as we are not looking at specific biological risk factors, we do not differentiate between sex and gender in our results and discussion.

For cross-sectional studies, we extracted study population demographics and outcome measures of each study area separately where available. For longitudinal studies, we extracted this information only at baseline. Data extraction was performed independently, and a small random proportion was performed in duplicate by four reviewers (DA, JC, HB and JT). The methodological quality of the included studies was also assessed using the Joanna Briggs Institute critical appraisal (assessment of risk of bias) checklist for studies that report prevalence data [13]. The critical appraisal checklist has nine criteria with options of Yes, No, Unclear or Not Applicable for each individual study. The global rating for each article was given based on the number of ‘Yes’ (0–3: poor quality, 4–7: moderate quality and 8–9: High quality). In studies where *M*:*F* prevalence of infection and/or infection intensity ratios were not provided directly, we calculated these using the data provided.

### Data synthesis

We did not carry out a meta-analysis on the intensity of infection *M*:*F* ratios for either *Schistosoma* species due to paucity of data. Amongst studies that report the mean intensity of infection, only 16 studies report the standard deviation of the mean intensity of infection in males and **females**. In addition to this, the studies differ in the way they calculate intensity of infection with some studies excluding zero counts and others including them. Instead, we present a descriptive summary of the results. In line with the aim of the paper, we provide an overview of the included studies and stratify results according to type of *Schistosoma* species and significance of the difference in *M*:*F* intensity of infection ratios. Statistical significance was defined as significance at 5% level as documented by all the individual studies. We also present a descriptive analysis of how risk factors varied between males and females.

We carried out a meta-analysis of the *M*:*F* prevalence of infection ratio to generate a pooled estimate for both *Schistosoma* species using the approach and adapted code provided by [14]. Heterogeneity was assessed using Higgin’s *I*^2^ statistic with *I*^2^>50% indicating significant heterogeneity [15]. The *R*^2^ (amount of heterogeneity accounted for by each factor or the combination of all significant factors) values were also calculated. For publication bias, we used Egger’s method [16] with significance p-value < 0.05. We employed the random-effects model for meta-analyses, weighting for the inverse of the variance using the R function ‘rma’ with the inverse of the variance of each study as the study weight. Some studies (cross-sectional) were entered into the model multiple times to account for multiple communities being reported. Using this function, we also conducted a meta-regression analysis with *M*:*F* prevalence ratios as the primary outcome of interest and age, quality rating, and baseline prevalence of the study area as the explanatory variables (S3 Text). Forest plots were created using the ‘forest’ function and all analyses were coded using R 4.0.1 [17].

## Results

### Overview of studies

**Fig 1:**
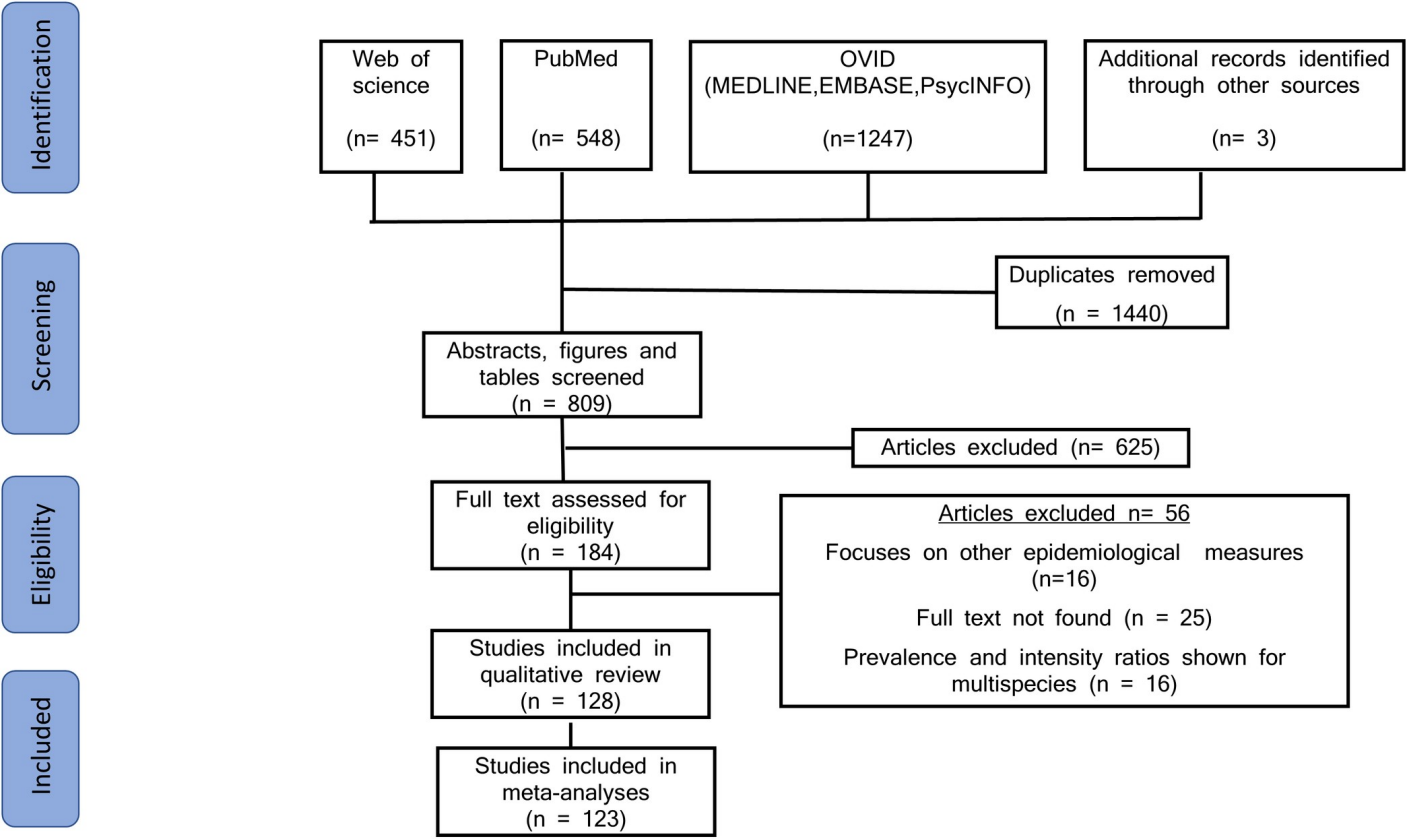
PRISMA diagram summarising inclusion and exclusion of all identified papers.

**Fig 2:**
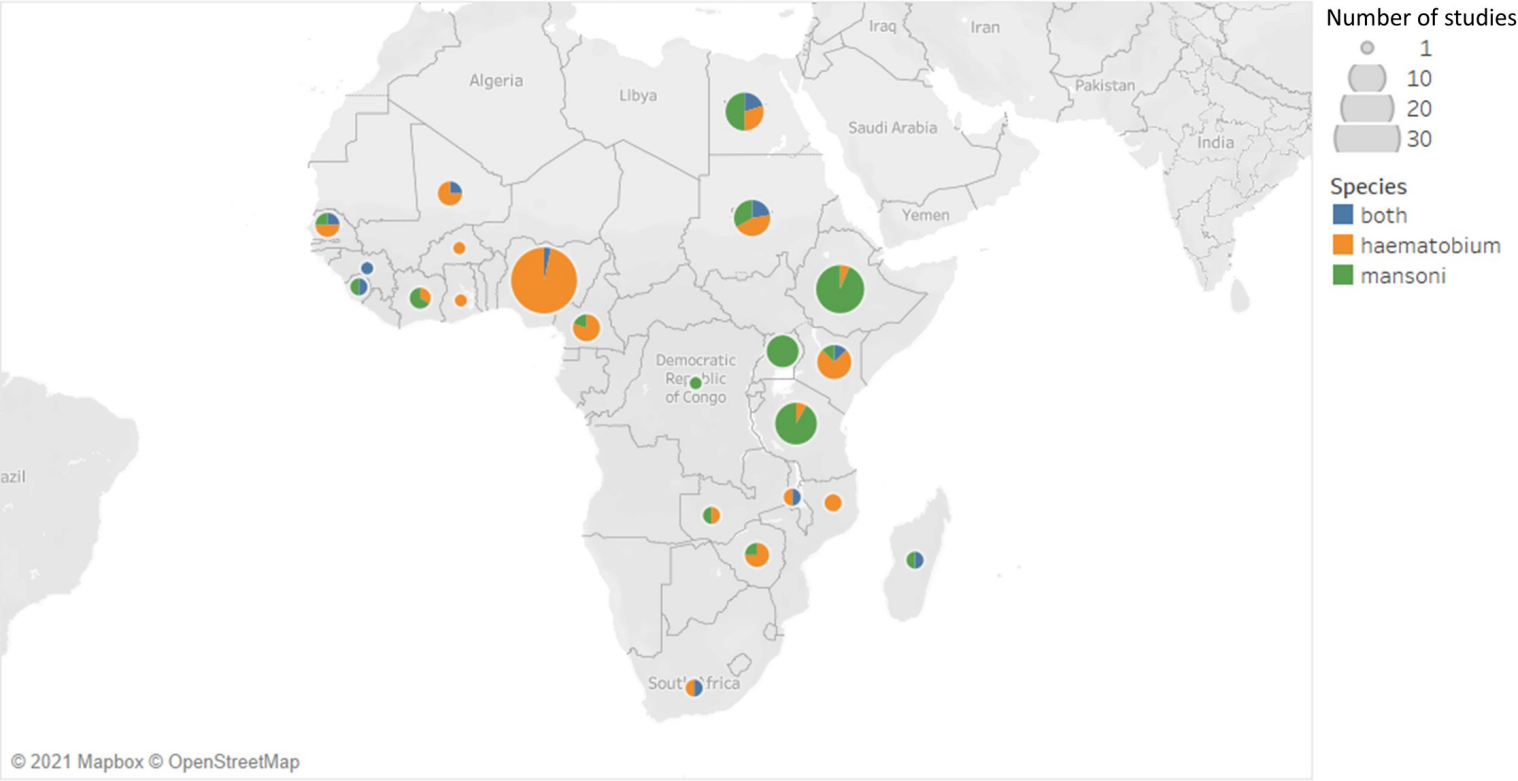
Map showing the geographical distribution across Africa of all papers included in this final review (n = 128). The size of each marker is proportional to the total number of studies conducted in each country and the colour corresponds to the species. This map was created using our data and the Tableau software [21]. Mapbox and OpenStreetMap are available by default in the Tableau Software Map Layers pane. Each Tableau software map built-in includes acknowledgements of Mapbox (https://www.mapbox.com/tableau/) and OpenStreetMap (https://www.openstreetmap.org/). OpenStreetMap is free to use under an open license.

**Fig 3:**
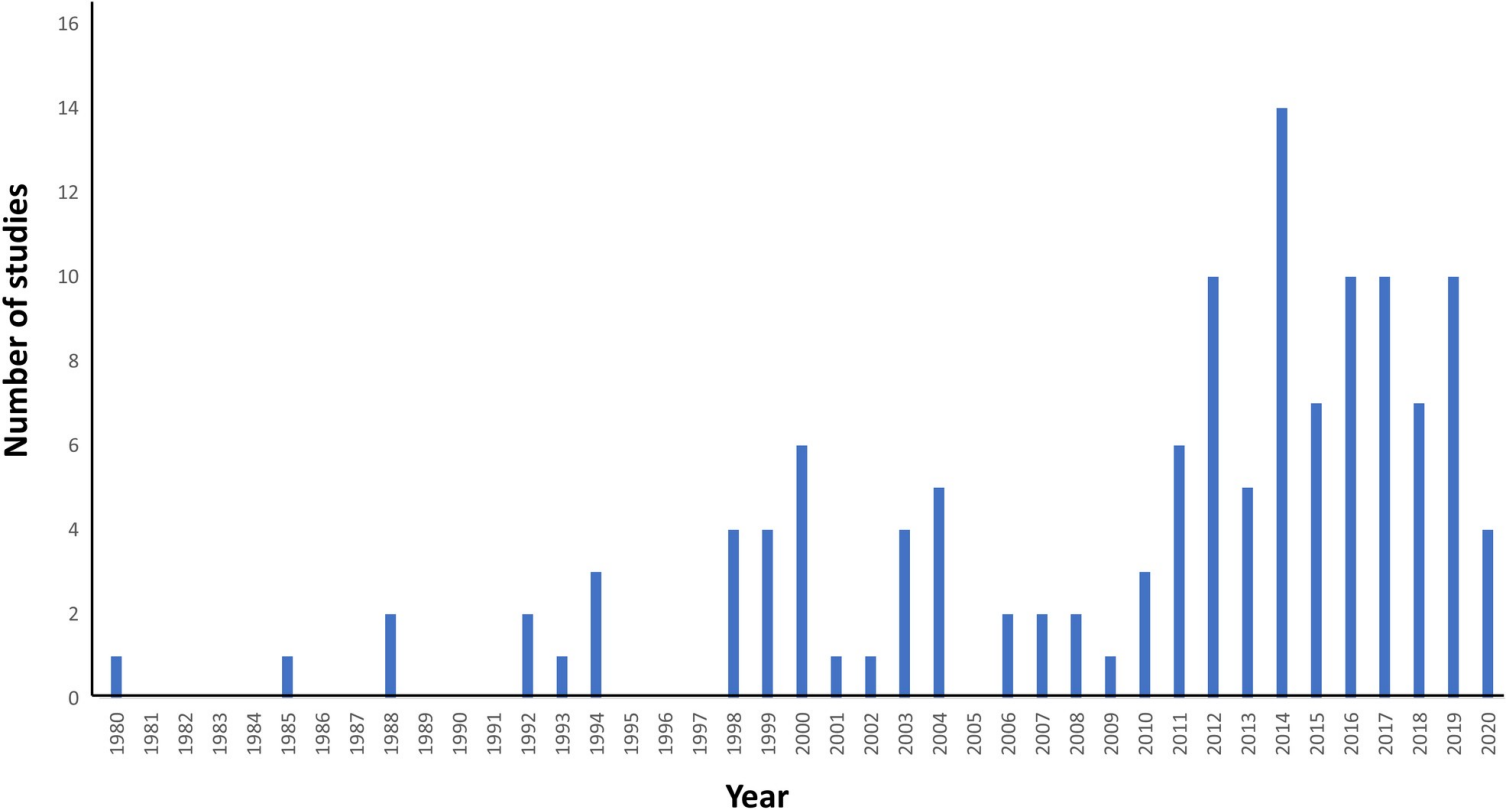
Distribution of publication year for the included papers (n = 128).

The search strategy yielded 2246 papers across the three databases, with four papers added from previous reviews. In addition to this we also had 3 papers from additional sources based on expert recommendation. After removing 1440 duplicates, 809 titles and abstracts were screened. After abstract screening, 625 further papers were excluded for reasons previously stated under Selection Criteria. The remaining 184 papers were assessed for full eligibility. Of these 184 papers, 56 papers were excluded because they did not match the inclusion criteria (Fig 1). A total of 128 papers were included in this systematic review. These 128 studies were identified from a total of 23 countries, with the majority undertaken in Nigeria (30 papers, 23.4%) and Ethiopia (16 papers, 12.5%) (Fig 2). Included papers were published between the years 1980 and 2020, with the majority published in the 21st century as most of the earlier published studies did not report sex-disaggregated prevalence and/or intensity data (Fig 3). 63 papers focused on *S*. *haematobium* only, while the remaining studies were focused on solely *S*. *mansoni* (51 papers) or both species (14 papers). The included studies covered a wide range of age groups including Pre-SAC, SAC and/or adults. One study indicated an age range of 0–91 years but no mean age [18]. Study sample size ranged from 83 [19] to 49,822 [20]. In total, there were 210,851 male participants and 195,988 female participants across the included studies. Most of the studies carried out statistical analysis on their data with respect to differences in male and females, mostly using a *χ*^2^ test for categorical variables.

### Quality of included studies

S2 Table provides the overall quality score for each study included in the review. Only 33 (around 25%) of the included studies met the threshold for high quality with eight studies obtaining the highest scores of 9/9 [22–29]. 85 studies were found to be of moderate quality. The remaining ten studies had poor quality particularly because there was a poor description of the study population (how and why participants were sampled, adequacy of sample size and coverage) and statistical methods.

### Summary of identified risk factors

Thirty-five of the included studies conducted a risk factor analysis to determine factors associated with *Schistosoma* infection. We summarise the risk factors found to be associated with *Schistosoma* infection, across the included studies (Table 1). These can be grouped into five major categories: (1) occupational and recreational water contact patterns such as fishing, swimming, laundry, farming [29–36]; (2) knowledge and beliefs such as education level [37,38]; (3) socio-economic factors such as income, occupation of parents [39–41]; (4) demographic factors such as age [30,35,42–49]; and (5) climatic and environmental factors such as availability of a nearby tap water source [50]. Two studies, one on *S*. *haematobium* in Zambia [51] and the other on *S*. *mansoni* in Uganda [52], found high altitudes to be significantly associated with higher infection rates, contrary to previous findings that climatic and environmental conditions at such high altitiudes are not favourable for schistosomiasis transmission [53,54]. This was attributed to snail abundance in temporary water bodies in both studies. Amongst the studies that report a significantly higher prevalence of infection in males, 16 studies [22,38,40,41,43,50,55–64], attribute this difference to gender-specific water-contact activities in which males tend to have more frequent water contact either because of cultural or religious beliefs, occupational reasons, or social roles.

**Table 1:** Risk factors and how they vary between males and females in included studies for *S*. *haematobium* and *S*. *mansoni*.

Risk factor	Males	Females
**(1) Occupational and recreational**	More males were reported to have water contact during activities such as swimming and agricultural activities and fishing [30–32,35,43,57,59] Farming as father’s occupation was significantly associated with higher infection rate [41]	Females were more involved in household duties [31] When women take up typical male roles such as fishing and farming, it increases their risk [58]
**(2) Knowledge and socio-cultural beliefs**	Male children considered haematuria (blood with urine) as a sign of maturity instead of a symptom of infection [38]; cultural practices predispose males to higher exposure [41,55]	Females were prohibited from bathing in open water sources due to religious beliefs [43]
	Increasing literacy level of family head (males or females) was associated with lower risk to infection [39]	
**(3) Demographic factors**	The male sex was found to be a significant risk factor associated with a higher risk of infection [38,40]	-
	Pre-SAC (≤ 5 *years*) males and females display almost similar water contact activities and many are infected when being washed or when accompanying their caregivers to contaminated water bodies [29,34] Young adults and the elderly were less likely to be infected than SAC [39]	
**(4) Socio-economic factors**	Low household income predisposed individuals to higher risk of infection [39]	
**(5) Climatic and environmental factors**	-	The absence of tap water predisposed females to higher risk as they are burdened with responsibility of fetching water from contaminated water sources [50]
	High altitudes were found to be significantly associated with higher infection rates in males and females [51]	

### S. haematobium results

Sex-disaggregated prevalence and/or intensity data for *S*. *haematobium* was reported by 77 papers. Five [36,65–68] of these papers do not report carrying out any statistical analysis on their data with respect to gender. Of the remaining 72 that do report this information, 70 report sex-disaggregated prevalence data, while only 41 report sex-disaggregated intensity of infection data. All the studies that reported differences which were found to statistically significant indicate that there is a higher prevalence and intensity of infection in males than females.

Of the 41 studies which reported their *M*:*F* intensity of infection ratios, these ratios ranged from 0.5 in a study in Sierra Leone [69] to 10.14 a study in Nigeria [41]. Only one of these extremes was statistically significant, with males showing a statistically higher intensity of infection [41]. In all the studies that reported a significant difference in intensity of infection between males and females, males were reported to have a higher mean intensity of infection.

Of the 70 studies which reported the *M*:*F* prevalence of infection ratios these ranged from 0.7 observed in a study in Zimbabwe [70] to 7.75 in a study in Nigeria [56]. While the difference was reported to be statistically significant in the Nigerian study with males being seven times more likely to be infected than females due to their water contact patterns, the study in Zimbabwe reported that the observed difference was not statistically significant. Most of the studies (76%) reported a sex ratio greater than 1, indicating a greater prevalence of infection in males than females although only 41% of these were reported to be statistically significant. Three studies [33,71,72] reported a significantly higher prevalence of infection in females. The age of the study participants in these three studies ranged from 4–21 years and so it is conceivable that differences in water contact patterns between males and females could explain this due to higher infection risk behaviours in young children. However, only one of the studies [33] reported this interaction and their results suggest that although within the study population, males had less prevalence of infection than females, females may have had less exposure (as measured by frequency of water contact) to the parasite than males.

**Fig 4:**
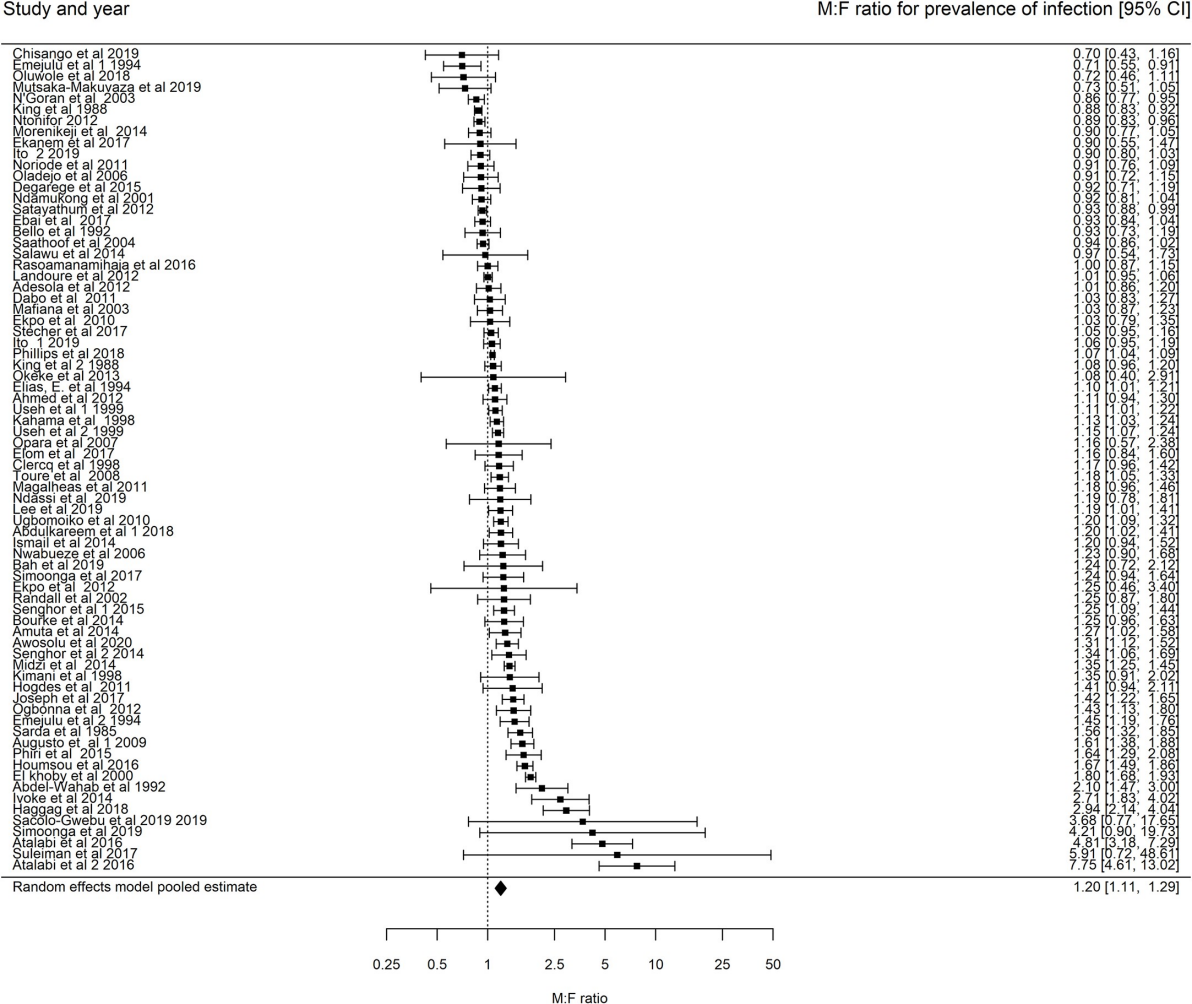
Forest plots showing the M:F prevalence of infection ratios and 95% CI for Schistosoma haematobium (74 communities and n = 71 studies, pooled M:F prevalence ratio is (1.19, 95% CI 1.11–1.29), heterogeneity: I^2^ = 95.95%). Points further to the right indicate higher prevalence in males.Analysis includes studies that report the number of males and females who were screened for S. haematobium infection and the fraction who tested positive regardless of the reported significance of the difference in M:F prevalence of infection ratios.

The random effects weighted model of *M*:*F* prevalence of infection ratios (including all studies regardless of whether the differences in prevalence of infection between males and females is reported to be significant or not) showed a significantly higher prevalence in males compared to females (1.20, 95% CI: 1.11–1.29) (Fig 4). The heterogeneity test statistic showed significant heterogeneity between publications *I*^2^ = 95.95%. As a result of such substantial heterogeneity, we conduct subgroup analysis to investigate what factors contribute to this heterogeneity. The univariate subgroup analysis (S3 Table) showed that the baseline prevalence, the lower age bound of the study participants and the sample size were significantly associated with *M*:*F* prevalence of infection ratios. The amount of heterogeneity contributed by the baseline prevalence, lower age bound of participants and sample size of study were 17.51%, 18.96% and 11.79% respectively (S3 Table). A larger sample size and a higher lower bound of the study participants each led to higher M:F prevalence of infection ratios (S3 and S4 Figs). On the other hand, a lower baseline prevalence leads to a higher *M*:*F* prevalence of infection ratios (S5 Fig).

### S. mansoni results

A total of 65 papers report sex-disaggregated prevalence and/or intensity of infection data for *S*. *mansoni*. Of these papers, 57 carried out statistical analyses relating to the differences in males and females. Of these 57 studies, 56 reported sex-disaggregated prevalence data, while only 35 reported sex-disaggregated intensity of infection data. 39% of the 56 studies reported a significant difference in prevalence of infection between males and females, while 31% of the 35 studies reported a significant difference in intensity of infection between males and females.

The *M*:*F* intensity of infection ratios in the 35 studies that reported it ranged from 0.55 to 2.79 observed in a study in Sudan [73] and a study in Uganda [23] respectively. Only one of these were statistically significant: the study in Uganda where males had on average three times higher egg counts than females. Males had a higher mean intensity of infection in all but one study that reported a significant difference in intensity of infection between males and females. This study was in a community in Sudan [64] where females had higher egg counts than males even though the observed prevalence was significantly higher in males. This was attributed to the duration spent on household chores such as laundry at the local canal by females in the study population.

The *M*:*F* prevalence of infection ratios in the 57 studies which reported these ratios ranged from 0.31 to 3.93 observed in a study in Sudan [73] and South Africa [49] respectively. Both studies report that the difference in prevalence between males and females was statistically significant. Females had a higher prevalence of infection than males in only three [73–75] of the studies. The ages of the participants in these studies ranged from 6 to above 40 (SAC and adults).

**Fig 5:**
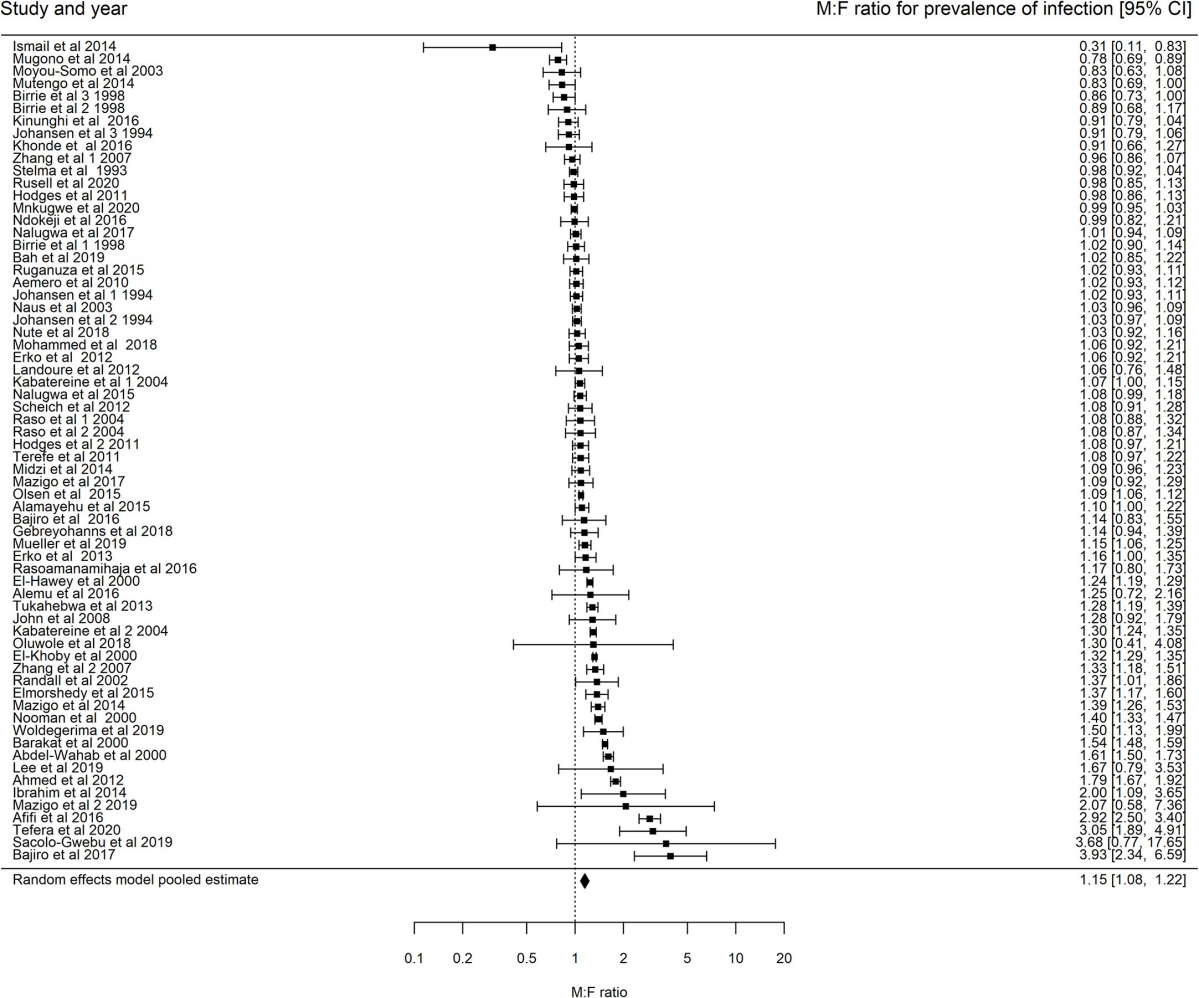
Forest plots showing the M:F prevalence of infection ratios and 95% CI for Schistosoma mansoni (66 communities and n = 61 studies, pooled M:F prevalence ratio is (1.15, 95% CI 1.08–1.22), heterogeneity: I^2^ = 96.43%). Points further to the right indicate higher prevalence in males. Analysis includes studies that report the number of males and females who were screened for S. mansoni infection and the fraction who tested positive regardless of the reported significance of the difference in M:F prevalence of infection ratio.

The random effects weighted model on *M*:*F* prevalence of infection ratios (including all studies regardless of whether the differences in prevalence of infection between males and females was reported to be significant or not) showed a significantly higher prevalence in males compared to females (1.15, 95% CI: 1.08–1.22; Fig 5). The heterogeneity test statistic test showed consistent heterogeneity of publications *I*^2^ = 96.43%. Due to the presence of high heterogeneity, subgroup analysis was performed to check for important contributing factors. The univariate subgroup analysis (S4 Table) showed that baseline prevalence of studies was significantly associated with *M*:*F* prevalence of infection ratios, while the other tested factors (sample size, lower and upper age limit of study participants) were not. The amount of heterogeneity contributed by the baseline prevalence was 10.5% (S4 Table). A higher baseline prevalence corresponds to a lower *M*:*F* prevalence of infection ratio (S6 Fig).

## Discussion

In this systematic review, we analysed studies reporting prevalence and intensity of *S*. *haematobium* and *S*. *mansoni* infection in Africa, quantifying the differences between males and females. We conducted a meta-analysis on the *M*:*F* prevalence of infection ratios obtained from the eligible studies, separately for both *Schistosoma* species using a random-effects model. We also conducted a univariate meta-regression analysis to determine what variables are associated with differences in male and female prevalence of infection. Our findings suggest that males are significantly more likely to be infected than females and that the higher the baseline prevalence, the lower the *M*:*F* prevalence of infection ratios for both *Schistosoma* species.

Our results suggest males are at an increased risk of exposure to contaminated water bodies. One plausible explanation is gender-related occupational roles, for example, in some of the studies, men spent more time fishing and practicing irrigation farming [30,42,73,76]. Also, in some study areas due to religious and sociocultural reasons, females are prohibited or at least discouraged from participating in activities such as swimming and fishing and are therefore exposed less often to infection [43,77]. Although females have some exposure activities which are particular to them, such as household chores, like washing dishes and doing laundry in contaminated water bodies, this seems to not be enough to increase their probability of infection beyond that of their male counterparts within the same communities. This has been attributed to the fact that their water contact activities involve the use of soap which may have a cercaricidal effect and thus reduce their risk whilst exposed to contaminated water [78]. There are, however, instances where this does not hold true—in a Kenyan study, a higher prevalence of infection in females was not directly linked to exposure patterns [33]—females had a significantly higher prevalence of *S*. *haematobium* infection even though males were reported to have more water contact.

It has been shown that males are less likely to participate in MDA programmes [79,80] and are thus may be be important maintainers of infections and transmission in some settings. Indeed the lower rate of male treatment, may in turn be contributing to their higher prevalence. We were unable to investigate this further here, but it is worth noting that the higher risk for males was documented before widespread MDA began from ~2002/3 indicating that even if this is involved it is unlikely to be a key driver. We also aimed to analyse the data pre and post MDA initiation, however as the timing of the study in relation to MDA was almost never reported, this was not possible. Our findings show that higher or lower prevalence of infection in one sex did not always translate to higher or lower mean egg counts in that sex when compared to the opposite sex. However, a paucity of adequately reported intensity sex dis-aggregated data meant we could not conduct a meta-analysis or regression on the intensity of infection ratios. Improvements to sex-disaggregated data reporting and susequent analysis is needed to elucidate this relationship. It is therefore evident that whilst water contact goes some way to explaining these sex-specific infection patterns, there are other risk factors to be considered.

An important factor this review cannot disentangle, is the difference in infection prevalence and/ or intensity in males and females by age. Results of the meta-regression analyses show that while the lower age limit of the study cohort was not significantly associated with higher *M*:*F* prevalence of infection for *S*. *mansoni*, it was for *S*. *haematobium*. Water-associated behaviors are possibly similar for very young males and females thereby leading to *M*:*F* prevalence of infection ratios closer to one. Whilst our initial aim had been to report data dis-aggregated by sex and age, we however found only 13 studies [20,23,29,44,45,55,81–87], that reported prevalence of infection data in males and females, stratified by age. This was not a sufficient number of studies, or a sufficient level of detail, to conduct a robust analysis. Additionally, the age stratifications vary greatly across studies, negatively impacting comparisons and analyses into the specific shape of the sex-age-infection profile. The risk of infection is generally believed to be higher for children, with infection intensity thought to peak between 10 and 20 years of age [88,89]. In conjunction with our results supporting higher infection risk for males, this highlights the necessity for studies to be designed with these age-varying patterns explicitly considered, and for these data to be reported in a consistent manner.

We note several limitations of this study. First of all, females were underrepresented, particularly in some studies [41,56,69,71,74,84,90,91] which may imply a sampling bias in the study designs themselves, which we could not overcome. Additionally, here we focus on infection intensity and infection prevalence, and do not investigate this relationship with the prevalence of morbidity, where there is some evidence to suggest in cases such as gential schistosomiasis, that females shoulder the burden of morbidity more so than males [92,93]. Along the same lines, the paucity of infection intensity data meant that we could not quantitatively investigate the relationship between host sex and infection intensity. These are important and undoubtedly interconnected points. In mixed sex studies, infection intensity and morbidity are not linearly related [94], but understanding sex differences across infection intensity and the manfestation of that as clinical morbidity will be vital to the understanding the distribution of the true, multidimensional burden of infection. Additionally, although we have focused our discussion on gender and the associated behavioural differences, we acknowledge that biological differences between the sexes may also contribute to the observed differences. For example different immune responses between the sexes have been argued to be an important factor in the observed differences in intensity of infection between males and females [95].

Some limitations related to data and study quality were also observed in the included studies. Using the Joanna Briggs Institute critical appraisal for studies that report prevalence data, showed that 72.9% of the studies had low to moderate quality. This was most often because numerous studies had either poorly conducted, poorly reported or completely un conducted/reported statistical analysis of prevalence and/or infection data in males and females. This was particularly evident in intensity of infection data as most studies did not report the standard deviation of the mean intensity of infection. Inappropriate analytical methods were more common in older studies, prior to the computational power for analysis we have now. Though it is worth mentioning that this was not always the case, and that unsuitable methods of analysis were used even in more recent works, presumably because the analyses are based on older papers/ methods. Alternative meta-analysis methods, like those making use of entire datasets could overcome these issues [96], though could also significantly limit the scope of the papers included if original datasets were not sharable (digitalised for example) or accessible. Papers also scored poorly for quality because a formal sample size calculation was rarely carried out.

We also observed some level of publication bias for studies on *S*. *haematobium*. Publication bias may exist when there is a preference to publish studies with significant findings. There is generally no preference to publish studies with specific *M*:*F* prevalence of infection ratios and in addition to this, not all subgroup analyses showed publication bias. Therefore, we believe that publication bias is unlikely to have distorted our results.

Given the high heterogeneity between the studies, the pooled *M*:*F* prevalence estimate should be interpreted with caution. Such variation may partially be attributed to factors including the baseline prevalence of the study areas, the age (lower age bound or upper age bound) of study participants or bias introduced by the sample size of each study. Based on the baseline prevalence, the pooled *M*:*F* prevalence of infection ratios decreased significantly from 1.34 and 1.25 in study areas with baseline prevalence less than 50% to 1.09 and 1.06 in study areas with baseline prevalence greater than 50% for *S*. *haematobium* and *S*. *mansoni* respectively. This may be because in areas of high endemicity, the infection is widespread such that the difference in prevalence between males and females is not so obvious. In relation to sample size and the lower age bound, it was observed that the larger the sample size or the higher the lower age bound of the studies, the higher the *M*:*F* prevalence of infection ratios and vice versa for *S*. *haematobium* only.

To conclude, our study highlights the importance of collecting, reporting and analysing unbiased sex-disaggregated data. Our results also expose a distinct lack of age by sex data collection and reporting [97]. Finally, through our quality assessment, we also highlight the need for improved statistical methods and reporting. Addressing these challenges will help to characterise the distribution of *Schistosoma* infection across demographic groups, which in turn will be vital for understanding how current intervention programmes can be improved to access and treat those most in need.

## Supporting information

S1 Table PRISMA guidelines completed checklist. (DOCX)

S2 Table All references included in the qualitative assessment of differences in infection prevalence and intensity between males and females as reported by the individual studies. Baseline prevalence is scored in keeping with the World Health Organization prevalence categories, where Mod is an abbreviation of moderate. Study size is broken down by males (M) and females (F). The quality scoring system follows that in the main text methods, with Poor = 0–3, medium = 4–7 (abbreviated as med here), and high = 8–9. *S*. *m* is the abbreviation for *Schistosoma mansoni*, nd *S*. *h* is the abbreviation for *Schistosoma haematobium*. Papers are listed in alphabetical order by title. Nr = Not reported.
(DOCX)

S3 Table Results of univariate meta-regression analysis showing the effect of age (lower and upper age limit of included studies), baseline prevalence and sample size on the *M:F* prevalence of infection ratio of *S. haematobium*. DF = degrees of freedom, * *depicts p-value < 0.05*.
(DOCX)

S4 Table Results of univariate meta-regression analysis showing the effect of age (lower and upper age limit of included studies), baseline prevalence and sample size on the *M:F* prevalence of infection ratio of *S. mansoni*. DF = degrees of freedom, * *depicts p-value < 0.05*.
(DOCX)

S1 Text Search strategy. (DOCX)

S2 Text Publication bias. (DOCX)

S3 Text Univariate meta-regression analysis. (DOCX)

S1 Fig Funnel plot for assessing publication bias in meta-analysis of 71 epidemiological studies in Africa of *S*. *haematobium*. The funnel graph shows the effect measure (*M*:*F* prevalence ratio) and standard error (S.E.) for each study.
(DOCX)

S2 Fig Funnel plot for assessing publication bias in meta-analysis of 61 epidemiological studies in Africa of *S*. *mansoni*. The funnel graph shows the effect measure (*M*:*F* prevalence ratio) and standard error (S.E.) for each study.
(DOCX)

S3 Fig Forest plots showing the *M*:*F* prevalence ratios and 95% CI for *S*. *haematobium* according to sample size; a) Studies with sample size less than 2251(mean sample size of included studies), pooled *M*:*F* prevalence ratio is 1.17 (95% *CI* 1.08−1.27), *I*^2^ = 93.15%, and b) studies with sample size greater than 2251; *M*:*F* prevalence of infection ratio is 1.31 (95% *CI* 1.08−1.59), *I*^2^ = 98%.
(DOCX)

S4 Fig Forest plots showing the *M*:*F* prevalence ratios and 95% CI for *S*. *haematobium* for studies where the lower age limit is greater than 5 years (21 communities and *n* = 19 studies, pooled *M*:*F* prevalence ratio is 1.54 (95% *CI* 1.24−1.91), heterogeneity: *I*^2^ = 97.85%). Analysis includes studies that report the number of individuals who were screened for *S*. *haematobium* infection and the fraction who tested positive distributed by sex regardless of the reported significance of the difference in *M*:*F* prevalence ratios.
(DOCX)

S5 Fig Forest plots showing the *M*:*F* prevalence ratios and 95% CI for *S*. *haematobium* according to baseline prevalence; a) Studies with baseline prevalence greater than 50% pooled *M*:*F* prevalence ratio is 1.09 (95% *CI* 1.03−1.15), *I*^2^ = 91.40%, and b) studies with baseline prevalence less than 50%; *M*:*F* prevalence of infection ratio is 1.34 (95% *CI* 1.16−1.55), *I*^2^ = 94.42%. Analyses includes studies that report the number of individuals who were screened for *S*. *haematobium* infection and the fraction who tested positive distributed by sex regardless of the reported significance of the difference in *M*:*F* prevalence ratios.
(DOCX)

S6 Fig Forest plots showing the *M*:*F* prevalence ratios and 95% CI for *S*. *mansoni* according to baseline prevalence; a) Studies with baseline prevalence greater than 50% pooled *M*:*F* prevalence ratio is 1.06 (95% *CI* 0.99−1.12), *I*^2^ = 90.38%, and b) studies with baseline prevalence less than 50%; *M*:*F* prevalence of infection ratio is 1.25 (95% *CI* 1.14−1.39), *I*^2^ = 97.84%.
(DOCX)
