# Supplementary material for: Gender-related differences in prevalence, intensity and associated risk factors of Schistosoma infections in Africa: A systematic review and meta-analysis
Source: PLoS Negl Trop Dis. 2021 Nov 17;15(11):e0009083. doi: 10.1371/journal.pntd.0009083 (PMC8635327; doi:10.1371/journal.pntd.0009083)
Supplement: S2 Table — Baseline prevalence is scored in keeping with the World Health Organization prevalence categories, where Mod is an abbreviation of moderate. Study size is broken down by males (M) and females (F). The quality scoring system follows that in the main text methods, with Poor = 0–3, medium = 4–7 (abbreviated as med here), and high = 8–9. S. m is the abbreviation for Schistosoma mansoni, nd S. h is the abbreviation for Schistosoma haematobium. Papers are listed in alphabetical order by title. Nr = Not reported. (DOCX) [file pntd.0009083.s002.docx]

***S2 Table***. All references included in the qualitative assessment of differences in infection prevalence and intensity between males and females as reported by the individual studies. Baseline prevalence is scored in keeping with the World Health Organization prevalence categories, where Mod is an abbreviation of moderate. Study size is broken down by males (M) and females (F). The quality scoring system follows that in the main text methods, with Poor = 0-3, medium = 4-7 (abbreviated as med here), and high = 8-9. *S. m* is the abbreviation for *Schistosoma mansoni*, nd *S. h* is the abbreviation for *Schistosoma haematobium*. Papers are listed in alphabetical order by title. Nr = Not reported.

| **Ref.** | **Author and year** | **Country** | **Year of publication** | **Study**  **time** | **Study**  **design** | **Species** | **Age**  **range** | **Baseline**  **prevalence** | **Study**  **size** | $\boldsymbol{M:F}$**Prevalence**  **ratios** | $\boldsymbol{M:F}$**Intensity ratios** | **Risk factors identified** | **Quality**  **score** |
| --- | --- | --- | --- | --- | --- | --- | --- | --- | --- | --- | --- | --- | --- |
| (1) | Phiri et al 2016 | Malawi | 2015 | 2005 | Cross-sectional | S. h | 1-na | Mod | M=733  F=909 | 1.64 | Nr | Bathing in the Shire river | Med |
| (2) | Rasoamanamihaja et al 2016 | Madagascar | 2016 | 2015 | Longitudinal | Both | 7-10 | Mod | S. h  M = 909  F = 1037  S. m  M=909  F=1025 | S. h  1  S. m  1.17 | Nr | Nr | High |
| (3) | Chisango et al 2019 | Zimbabwe | 2019 | Nr | Longitudinal | S. h | 7-13 | Mod | M = 105  F=107 | 0.70 | Nr | Nr | Poor |
| (4) | Elmorshedy et al 2015 | Egypt | 2015 | 2011 | Cross-sectional | S. m | <5-40+ | Mod | M=690  F=803 | 1.97 | Nr | Nr | Med |
| (5) | King et al 1988 | Kenya | 1988 | 1984 | Longitudinal | S. h | 4-21 | High | M=1448  F=1180 | 0.88 | 1 | Nr | Med |
| **Ref.** | **Author and year** | **Country** | **Year of publication** | **Study**  **time** | **Study**  **design** | **Species** | **Age**  **range** | **Baseline**  **prevalence** | **Study**  **size** | $\boldsymbol{M:F}$**Prevalence**  **ratios** | $\boldsymbol{M:F}$**Intensity ratios** | **Risk factors identified** |  |
| (6) | Abebe et al 2014 | Ethiopia | 2014 | 2012 | Cross-Sectional | S. m | <10-30+ | High | M=211  F=160 | 1 | 1 | Location specific risk. | Med |
| (7) | Mohammed et al 2018 | Ethiopia | 2018 | 2016-17 | Cross-sectional | S. m | 5-72 | High | M=413  F=598 | 1.05 | Nr | Lack of knowledge regarding epidemiology and prevention, regular use of river. | Med |
| (8) | Mazigo et al 2014 | Tanzania | 2014 | 2012 | Cross-sectional | S. m | 21-55 | Mod | M=840  F=945 | 1.38 | 1.8 | Young, male, fishing, location specific risk, illiteracy. | High |
| (9) | Ogbonna et al 2012 | Nigeria | 2012 | 2006-7 | Cross-sectional | S. h | 4-68 | Mod | M=636  F=701 | 1.43 | 1.03 | Nr | Med |
| (10) | Russell et al 2020 | Madagascar | 2020 | 2016 | Cross-sectional | S. m | 5-14 | High | M=134  8=141 | 0.98 | Nr | Working in rice paddies | High |
| (11) | Morenikeji et al 2014 | Nigeria | 2014 | Nr | cross-sectional | S. h | 3-15+ | High | M=255  F=232 | 0.90 | 1 | Nr | Med |
| (12) | Hodges et al 2011 | Guinea | 2011 | Nr | cross-sectional | Both | 9-14 | Low | M=262  F=158 | S. h  1.41  S. m  0.99 | S. h  1.9  S. m  1.12 | Nr | High |
| (13) | Bourke et al 2014 | Zimbabwe | 2014 | ongoing | Cross-sectional | S. h | 4-84 | Mod | M=94  F=101 | 1.25 | Nr | Nr | Med |
| **Ref.** | **Author and year** | **Country** | **Year of publication** | **Study**  **time** | **Study**  **design** | **Species** | **Age**  **range** | **Baseline**  **prevalence** | **Study**  **size** | $\boldsymbol{M:F}$**Prevalence**  **ratios** | $\boldsymbol{M:F}$**Intensity ratios** | **Risk factors identified** |  |
| (14) | Kahama et al 1998 | Kenya | 1998 |  |  | S. h | 6-17 | High | M=252  F=218 | 1.13 | 1.82 | Nr | High |
| (15) | Midzi et al 2014 | Zimbabwe | 2014 | 2010-2011 | cross-sectional | S. h | 10-15 | Low | M=6417  F=6620 | 1.35 | Nr | Nr | High |
| (16) | Joseph et al 2017 | Nigeria | 2017 | Nr | Cross-sectional | S. h | ≥20 | Low | M=2256  F=1501 | 1.42 | 1.06 | Nr | Med |
| (17) | Ebai et al 2017 | Cameroon | 2017 | 2014 | Longitudinal | S. h | 4-76 | Mod | M=100  F=100 | 0.93 | Nr | Nr | Poor |
| (18) | Raso et al 2004 | Cote d'Ivoire | 2004 | 2002 | cross-sectional | S. m | 5d -91y | Mod | M=278  F=267 | 1.08 | Nr | Nr | Med |
| (19) | Erko et al 2012 | Ethiopia | 2012 | 2010 | cross-sectional | S. m | 6-22 | High | M=179  F=120 | 1.05 | Nr | Nr | Poor |
| (20) | N’Goran et al 2003 | Cote d'Ivoire | 2003 | 2000 |  | S. h | 5-15 | High | M=201  F=153 | 0.86 | Nr | Nr | Med |
| (21) | Senghor et al 2015 | Senegal | 2015 | 2011 | longitudinal | S. h | 5-15 | High | M=187  F=142 | 1.25 | 3.07 | Nr | Poor |
| (22) | Haggag et al 2018 | Egypt | 2018 | 2016-2017 |  | S. h | 6-16 | Low | M=21660  F=8423 | 2.9 | 1.14 | Nr | Med |
| **Ref.** | **Author and year** | **Country** | **Year of publication** | **Study**  **time** | **Study**  **design** | **Species** | **Age**  **range** | **Baseline**  **prevalence** | **Study**  **size** | $\boldsymbol{M:F}$**Prevalence**  **ratios** | $\boldsymbol{M:F}$**Intensity ratios** | **Risk factors identified** |  |
| (23) | Ntonifor et al 2012 | Cameroon | 2012 | 2011 | Longitudinal | S. h | 5-16 | High | M=302  F=302 | 0.89 | Nr | Nr | Poor |
| (24) | Lee et al 2019 | Sudan | 2019 | Nr | Cross-sectional | Both | ≤6-30+ | Mod | S. h  M=362  F=771  S. m  M=333  F=705 | S. h  1.44  S. m  1.67 | S. h  1.29  S. m  Nr | Age and Sex | Poor |
| (25) | Clercq et al 1999 | Senegal | 1999 | 1996-1997 | Community | Both | 1-40+ | S. h  Mod  S. m  High | Nr | S. h  0.89  S. m  1.04 | S. h  0.51  S. m  0.69 | Nr | Med |
| (26) | Tefera et al 2020 | Ethiopia | 2020 | 2017 | cross-sectional | S. m | 7-17 | Mod | M=196  F=132 | 3.04 | Nr | Male, bare foot river crossings, recreational use of nearby river | Med |
| (27) | Stelma et al 1993 | Senegal | 1993 | 1991 | Community | S. m | 1-40+ | High | M=188  F=234 | 0.98 | 1.01 | Nr | Med |
| **Ref.** | **Author and year** | **Country** | **Year of publication** | **Study**  **time** | **Study**  **design** | **Species** | **Age**  **range** | **Baseline**  **prevalence** | **Study**  **size** | $\boldsymbol{M:F}$**Prevalence**  **ratios** | $\boldsymbol{M:F}$**Intensity ratios** | **Risk factors identified** |  |
| (28) | Afifi et al 2015 | Sudan | 2015 | 2013 | cross-sectional | S. m | 1-50+ | Nr | M=1195  F=1238 | 2.92 | 0.64 | Nr | High |
|  |  |  |  |  |  |  |  |  |  |  |  |  | **Ref.** |
| (29) | Abdel-wahab 1 et al 2020 | Egypt | 2000 | 1992 | cross-sectional | Both | 0-70+ | Low | M=2556  F=2658 | Nr | Nr | Nr | Med |
| (30) | El-Hawey et al 2000 | Egypt | 2000 | Nr | cross-sectional | S. m | 0-55+ | Mod | M=5628  F=6048 | 1.24 | Nr | Nr | Med |
| (31) | Nooman et al 2000 | Egypt | 2000 | Nr | cross-sectional | S. m | 0-65+ | Mod | M=3755  F=4035 | 1.40 | Nr | males washing in the canals, women washing clothes and utensils in the canals, children playing in canals and being 15 years old. | Med |
| **Ref.** | **Author and year** | **Country** | **Year of publication** | **Study**  **time** | **Study**  **design** | **Species** | **Age**  **range** | **Baseline**  **prevalence** | **Study**  **size** | $\boldsymbol{M:F}$**Prevalence**  **ratios** | $\boldsymbol{M:F}$**Intensity ratios** | **Risk factors identified** |  |
| (32) | Abdel-wahab et al 2000 | Egypt | 2000 | Nr | cross-sectional | S. m | 0-60+ | Low | M=4065  F=4059 | 1.61 | Nr | age>10 years old, male, living in ezbas, males bathing in, women, washing clothing or utensils in, or children swimming or playing in canals. A history of schistosomiasis, and recent his-tory of blood in the stool | High |
| (33) | Barakat et al 2000 | Egypt | 2000 | Nr | cross-sectional | S. m | 0-60+ | Mod | M=7288  F=7741 | 1.54 | 1.31 | <15 playing in canals, exposure to canal water, previous infection. | High |
| (34) | El-Khoby et al 2000 | Egypt | 2000 | Nr | cross-sectional | Both | 0-60+ | Mod | S. h  M=18939  F=20292  S. m  M=24240  F=25582 | S. h  1.8  S. m  1.32 | Nr | Haematobium  burning micturition  Mansoni  Children, bloody in stool  Both  Exposure to canal water, living in Ezbas, history of treatment. | high |
| **Ref.** | **Author and year** | **Country** | **Year of publication** | **Study**  **time** | **Study**  **design** | **Species** | **Age**  **range** | **Baseline**  **prevalence** | **Study**  **size** | $\boldsymbol{M:F}$**Prevalence**  **ratios** | $\boldsymbol{M:F}$**Intensity ratios** | **Risk factors identified** |  |
| (35) | Birrie et al 1998 | Ethiopia | 1998 | 1994 | cross-sectional | S. m | 0-40+ | Mod | Kemise  M=388  F=363  Harbu  M=222  F=224  Bati  M=283  F=333 | Kemise  1.01  Harbu  0.89  Bati  0.86 | Kemise  0.98  Harbu  1.02  Bati  0.75 | Nr | Med |
| (36) | Sulieman et al 2017 | Sudan | 2017 | 2016 | Cross-sectional | S. h | 7-14 | Low | M=194  F=191 | 5.91 | 1.57 | Swimming and working in fields | Med |
| (37) | Ibrahim et al 2014 | Sudan | 2014 | 2011-12 | cross-sectional | S. m | 6-16 | Low | M=124  F=90 | 2 | Nr | Nr | Med |
| (38) | Ugbomoiko et al 2010 | Nigeria | 2010 | 2006-2007 | cross-sectional | S. h | 2-78 | High | M=545  F=478 | 1.20 | 1.54 | low family income, number of children aged 10-15 living in the household, not living with biological parents, living close to a local river. | Med |
| **Ref.** | **Author and year** | **Country** | **Year of publication** | **Study**  **time** | **Study**  **design** | **Species** | **Age**  **range** | **Baseline**  **prevalence** | **Study**  **size** | $\boldsymbol{M:F}$**Prevalence**  **ratios** | $\boldsymbol{M:F}$**Intensity ratios** | **Risk factors identified** | **Ref.** |
| (39) | Satayathum et al 2012 | Kenya | 2012 | 1984-1992 | Longitudinal | S. h | 5-20 | High | M=2473  F=2367 | 0.93 | Nr | Water contact | Med |
| (40) | Opara et all 2007 | Nigeria | 2007 | 2005 | cross-sectional | S. h | 0-5 | Low | M=81  F=55 | 1.16 | 1.11 | Nr | Med |
| (41) | Scheich et al 2012 | Tanzania | 2012 | 2007 | cross-sectional | S. m | 6-17 | High | M=180  F=180 | 1.08 | 1.42 | Nr | Medium |
| (42) | Ahmed et al 2012 | Sudan | 2012 | 2007-2008 | longitudinal | S. m | 6-15 | High | M=1488  F=1253 | 1.79 | 1.75 | Nr | Med |
| (43) | Mutengo et al 2014 | Zambia | 2014 | Nr | cross-sectional | S. m | 7-50 | Mod | M=285  F=469 | 0.84 | 0.96 | Nr | Med |
| (44) | Ito et al 2019 | Nigeria | 2019 | 2015 | Cross-sectional | S. h | 5-13 | High | M=529  F=315 | Aviara  1.06  Igbide  0.9 | Nr | Nr | Med |
| (45) | Hodges et al 2 2011 | Sierra Leone | 2011 | 2009 | cross-sectional | S. m | 9-14 | Mod | M=905  F=855 | 1.08 | 1.02 | Nr | High |
| (46) | Augusto et al 2014 | Mozambique | 2009 | 2004 | longitudinal | S. h | 8-16 | Mod | M=229  F=301 | 1.61 | 1.34 | Nr | High |
| **Ref.** | **Author and year** | **Country** | **Year of publication** | **Study**  **time** | **Study**  **design** | **Species** | **Age**  **range** | **Baseline**  **prevalence** | **Study**  **size** | $\boldsymbol{M:F}$**Prevalence**  **ratios** | $\boldsymbol{M:F}$**Intensity ratios** | **Risk factors identified** | **Ref.** |
| (47) | Nalugwa et al 2015 | Uganda | 2015 | 2012-2013 | longitudinal | S. m | 1-5 | Mod | M=1545  F=1513 | 1.08 | 1.17 | Increased location specific risk with increased age, use of the lake, duration of lake contact | High |
| (48) | Mugono et al 2014 | Tanzania | 2014 | Nr | cross-sectional | S. m | 4-15 | High | M=353  F=421 | 0.94 | 1.28 | Duration of lake contact and parental occupation | Med |
| (49) | Mueller et al 2019 | Tanzania | 2019 | Nr | cross-sectional | S. m | 1-95 | High | M=417  F=513 | 1.15 | 1.1 | Being male, risk associated with specific age groups | High |
| (50) | Emejulu et al 1994 | Nigeria | 1994 | 1990-1992 | cross-sectional | S. h |  | Mod | Agulu  M=140  F=120  Nri  M=200  F=220 | Agulu  0.71  Nri  1.45 | Nr  Nr | Nr  Nr | Poor |
| (51) | Magalhaes et al 2011 | Ghana | 2011 | 2008 | modelling | S. h | 5-19 | Low | M=2209  F=2236 | 1.18 | Nr | Nr | High |
| **Ref.** | **Author and year** | **Country** | **Year of publication** | **Study**  **time** | **Study**  **design** | **Species** | **Age**  **range** | **Baseline**  **prevalence** | **Study**  **size** | $\boldsymbol{M:F}$**Prevalence**  **ratios** | $\boldsymbol{M:F}$**Intensity ratios** | **Risk factors identified** | **Ref.** |
| (52) | Mwandawiro et al 2013 | Kenya | 2013 | 2012 | cross-sectional | S. m | 3-21 | Low | S. h  M~9980  F~9840  S. m  M~9980  F~9840 | S. h  1  S. m  1 | S. h  2.67  S. m  1 | Nr | High |
| (53) | Raso et al 2 2004 | Cote d'Ivoire | 2004 | Nr | cross-sectional | S. m | 0-91 | Mod | M=249  F=251 | 1.08 | Nr | Nr | Med |
| (54) | Moyou-somo et al 2003 | Cameroon | 2003 | 2001 | cross-sectional | S. m | <5-20+ | Mod | M=93  F=87 | 0.83 | 1.15 | Nr | Poor |
| (55) | Stecher et al 2017 | Mali | 2017 |  | longitudinal | S. h | 2-40 | High | M=212  F=189 | 1.05 | 1.06 | Nr | High |
| (56) | Mwabueze et al 2007 | Nigeria | 2006 | 2003-2005 | longitudinal | S. h | 5-12 | Low | M=300  F=280 | 1.23 | 2.26 | Nr | Poor |
| **Ref.** | **Author and year** | **Country** | **Year of publication** | **Study**  **time** | **Study**  **design** | **Species** | **Age**  **range** | **Baseline**  **prevalence** | **Study**  **size** | $\boldsymbol{M:F}$**Prevalence**  **ratios** | $\boldsymbol{M:F}$**Intensity ratios** | **Risk factors identified** | **Ref.** |
| (57) | Zhang et al 2007 | Uganda | 2007 | Nr | longitudinal | S. m | 6-11 | Mod | Community 1 M=879  F=821  Community 2  M=441  F=413 | 0.96  1.34 | 1.13  2.24 | Nr | High |
| (58) | Randall et al 2002 | Malawi | 2002 | 1999 | Cross-sectional | Both | 9-16 | Mod | S. h  M=232  F=240  S. m  M=224  F=232 | S. h  1.25  S. m  1.378 | S. h  2  S. m  1.49 | Nr | Poor |
| (59) | Saathoff et al 2004 | South Africa | 2004 | 1998 | Cross-sectional | S. h | <9-15+ | High | M=510  F=599 | 0.94 | 0.93 | Nr | Med |
| (60) | Mnkugwe et al 2020 | Tanzania | 2020 | 2017 | Cross-sectional + Longitudinal | S. m | 7-19 | High | M=413  F=417 | 0.99 | 1.22 | Younger children more prone to infection | High |
| **Ref.** | **Author and year** | **Country** | **Year of publication** | **Study**  **time** | **Study**  **design** | **Species** | **Age**  **range** | **Baseline**  **prevalence** | **Study**  **size** | $\boldsymbol{M:F}$**Prevalence**  **ratios** | $\boldsymbol{M:F}$**Intensity ratios** | **Risk factors identified** | **Ref.** |
| (61) | Atalabi et al 2016 | Nigeria | 2016 | 2015 | Cross-sectiona | S. h | 10-23 | Mod | M=401  F=317 | 4.81 | 10.14 | Fathers occupation, sex and age | Med |
| (62) | Ndokeji et al 2016 | Tanzania | 2016 | Nr | Cross-sectional | S. m | 4-14 | High | M=215  F=239 | 0.99 | Nr | Use of the lake for occupational and recreational reasons | High |
| (63) | Mazigo et al 2 2019 | Tanzania | 2019 | 2017 | Cross-sectional | S. m | 1-16 | Mod | M=58  F=45 | 2.07 | Nr | Nr | High |
| (64) | Bajiro et al 2017 | Ethiopia | 2017 | 2014 | Cross-sectional | S. m | 5-19 | Low | M=501  F=499 | 3.93 | Nr | Nr | Med |
| (65) | Senghor et al 2 2014 | Senegal | 2014 | 2009 | Cross-sectional | S. h | 7-15 | Mod | M=101  F=109 | 1.34 | 2 | Nr | Med |
| (66) | Ndamukong et al 2001 | Cameroon | 2001 | Nr | Cross-sectional | S. h | 5-16 | High | M=90  F=85 | 0.92 | 1.04 | Nr | Poor |
| (67) | Woldegerima et al 2019 | Ethiopia | 2019 | 2017 | Cross-sectional | S. m | 9-14 | Mod | M=183  F=189 | 1.5 | Nr | Nr | High |
| (68) | Ndassi et al 2019 | Cameroon | 2019 | 2017 | Cross-sectional | S. h | 3-62 | Low | M=433  F=568 | 1.19 | Nr | Nr | Med |
| **Ref.** | **Author and year** | **Country** | **Year of publication** | **Study**  **time** | **Study**  **design** | **Species** | **Age**  **range** | **Baseline**  **prevalence** | **Study**  **size** | $\boldsymbol{M:F}$**Prevalence**  **ratios** | $\boldsymbol{M:F}$**Intensity ratios** | **Risk factors identified** | **Ref.** |
| (69) | Sacolo-Gwebu et al 2018 | South Africa | 2019 | 2018 | Cross-sectional | Both | 1-5 | Low | M=557  F=586 | S. h  3.68  S. m  3.68 | Nr | Age, locality and frequency of visits to stream | High |
| (70) | Ivoke et al 2014 | Nigeria | 2014 | 2012 | Cross-sectional | S. h | 6-20 | Mod | M=527  F=367 | 2.71 | Nr | Caregivers age and habit of bathing children in river water. Source of domestic water. | Med |
| (71) | Gebreyohanns et al 2018 | Ethiopia | 2018 | 2017 | Cross-sectional | S. m | 5-70 | Mod | M=242  F=169 | 1.14 | Nr | Age, using and duration of use of local water source for recreational and domestic purposes. Open shoes. | High |
| (72) | Degarege et al 2015 | Ethiopia | 2015 | 2014 | Cross-sectional | S. h | 5-16 | Mod | M=507  F=378 | 0.92 | 0.73 | Nr | Med |
| (73) | Bajiro et al 2016 | Ethiopia | 2016 | 2014 | Cross-sectional | S. m | 6-18 | Mod | M=238  F=262 | 1.14 | Nr | Nr | Med |
| (74) | Khonde et al 2016 | DRC | 2016 | 2016 | Cross-sectional | S. m | 10-18 | Mod | M=212  F=176 | 0.91 | Nr | Nr | Med |
| (75) | Nute et al 2018 | Ethiopia | 2018 | 2011-2015 | Cross-sectional | S. m | 6-15 | Low | M=7403  F=8052 | 1.03 | Nr | Nr | Med |
| **Ref.** | **Author and year** | **Country** | **Year of publication** | **Study**  **time** | **Study**  **design** | **Species** | **Age**  **range** | **Baseline**  **prevalence** | **Study**  **size** | $\boldsymbol{M:F}$**Prevalence**  **ratios** | $\boldsymbol{M:F}$**Intensity ratios** | **Risk factors identified** | **Ref.** |
| (76) | Oluwole et al 2018 | Nigeria | 2018 | 2013 | Cross-sectional | Both | 5-14 | Low | M=1114  F=1034 | S. h  0.72  S. m  1.3 | S. h  1.17  S. m  0.33 | Domestic and recreational use of lakes, rivers and streams. | Med |
| (77) | Amuta et al 2014 | Nigeria | 2014 | 2012 | Cross-sectional | S. h | 1-15 | High | M=-170  F=130 | 1.27 | 1.29 | Nr | Med |
| (78) | Ismail et al 2014 | Sudan | 2014 | 2009/10 |  | Both | 7-15 | S. h  Mod  S. m  Low | M=176  F=162 | S. h  1.2  S. m  0.61 | S. h  1.13  S. m  0.55 | Irrigation and swimming practices | Med |
| (79) | Naus et al 2003 | Uganda | 2003 | 1996 | Community | S. m | 5-59 | High | M=168  F=210 | 1.02 | Nr | Nr | Med |
| (80) | Clercq et al 1998 | Mali | 1998 | Nr | Cross-sectional | S. h | 6-11 | Mod | M=275  F=262 | 1.17 | Nr | Nr | Med |
| (81) | Kimani et al 2018 | Kenya | 2018 | Nr | Cross-sectional | S. h | 0-72 m | Mod | M=205  F=195 | 1.35 | 1.85 | Nr | High |
| **Ref.** | **Author and year** | **Country** | **Year of publication** | **Study**  **time** | **Study**  **design** | **Species** | **Age**  **range** | **Baseline**  **prevalence** | **Study**  **size** | $\boldsymbol{M:F}$**Prevalence**  **ratios** | $\boldsymbol{M:F}$**Intensity ratios** | **Risk factors identified** | **Ref.** |
| (82) | Abdel-wahab et al 1980 | Egypt | 1992 | Nr | Community | S. h | 12-16 | Mod | M=274  F=148 | 2.10 | Nr | Nr | Med |
| (83) | Elias et al 1994 | Sudan | 1994 | 1989 | Cross-sectional | S. h | <7-40+ | Mod | M=2490  F=2235 | 1.1 | Nr | Nr | High |
| (84) | Ahmed et al 2012 | Sudan | 2012 | 2008-2090 | Longitudinal | S. h | 1-16 | High | M=309  F=253 | 1.11 | 1.37 | Nr | Med |
| (85) | Ekpo et al 2012 | Nigeria | 2012 | Nr | Cross-sectional | S. h | ≥6 | Mod | M=49  F=34 | 1.25 | Nr | Nr | Med |
| (86) | Bello et al 1992 | Nigeria | 1992 | Nr | Cross-sectional | S. h | 5-18 | Mod | M=331  F=94 | 0.93 | Nr | Nr | Med |
| (87) | Ruganuza et al 2015 | Tanzania | 2015 | Nr | Cross-sectional | S. m | 1-6 | Mod | M=197  F=203 | 1.02 | 1.17 | Nr | Med |
| (88) | Alemu et al 2016 | Ethiopia | 2016 | 2015 | Cross-sectional | S. m | 6m-6y | Mod | M=183  F=218 | 1.25 | Nr | Nr | Med |
| (89) | Mazigo et al 2017 | Tanzania | 2017 | 2013 | Cross-sectional | S. m | 18-89 | High | M=224  F=188 | 1.09 | 1.26 | history of water contact and living at a distance of less than 500 m from the lake water contact point | Med |
| (90) | Alamayehu et al 2015 | Ethiopia | 2015 | 2014 | Cross-sectional | S. m | 5-19 | High | M=201  F=183 | 1.10 | 1.72 | Nr | High |
| **Ref.** | **Author and year** | **Country** | **Year of publication** | **Study**  **time** | **Study**  **design** | **Species** | **Age**  **range** | **Baseline**  **prevalence** | **Study**  **size** | $\boldsymbol{M:F}$**Prevalence**  **ratios** | $\boldsymbol{M:F}$**Intensity ratios** | **Risk factors identified** | **Ref.** |
| (91) | Nalugwa et al 2017 | Uganda | 2017 | 2013-2014 | Cross-sectional | S. m | 1-5 | High | M=461  F=455 | 0.93 | Nr | Nr | Med |
| (92) | Bah et al 2019 | Sierra Leone | 2019 | 2009,2010,2014 and 2016 | Longitudinal and cross sectional | Both | 9-14 | S. h  Mod  S. m  Low | S. h  M=1844  F=1788  S. m  M=1530  F=1453 | S. h  1.02  S. m  1.24 | S. h  0.80  S. m  0.5 | Nr | Med |
| (93) | Terefe et al 2011 | Ethiopia | 2011 | 2007 | Cross-sectional | S. m | 5-20+ | High | M=218  F=201 | 1.08 | 1.91 | Nr | Med |
| (94) | Abdel-wahab et al 1980 | Egypt | 1980 | 1977 | Cross-sectional | S. m | ≥1 | High | M=325  F=212 | 2.05 | 1.45 | Nr | Med |
| (95) | John et al 2008 | Uganda | 2008 | Nr | Cross-sectional | S. m | 6-18 | Mod | M=189  F=181 | 1.14 | 1.59 | Nr | High |
| (96) | Landoure et al 2012 | Mali | 2012 | 2004/2010 | Cross-sectional | Both | 7-14 | S. h  High  S. m  Mod | M=326  F=322 | S. h  1.01  S. m  1.05 | S. h  1.13  S. m  2.23 | Use of Crater water | Med |
| **Ref.** | **Author and year** | **Country** | **Year of publication** | **Study**  **time** | **Study**  **design** | **Species** | **Age**  **range** | **Baseline**  **prevalence** | **Study**  **size** | $\boldsymbol{M:F}$**Prevalence**  **ratios** | $\boldsymbol{M:F}$**Intensity ratios** | **Risk factors identified** | **Ref.** |
| (97) | Aemero et al 2014 | Ethiopia | 2014 | 2010 | Cross-sectional | S. m | 5-49+ | Mod | M=548  F=545 | 1.02 | Nr | Nr | Med |
| (98) | Johansen et al 1994 | Kenya | 1994 | Nr | Cross-sectional | S. m | 9-16 | High | Miu  M=76  F=83  Kitengei  M=82  F=78  Misuuni  M=55  F=44 | Miu  1.02  Kitengei  1.03  Misuuni  0.99 | Miu  1.95  Kitengei  1.48  Misuuni  1 | Nr | Med |
| (99) | Mohamed-Ali et al 1999 | Sudan | 1999 | Nr | Community | S. m | <6-50+ | Nr | Nr | 1.07 | Nr | Nr | Med |
| (100) | Toure et al al | Burkina Faso | 2008 | 2005-2007 | longitudinal Cohort study | S. h | 6-14 | High | M=403  F=360 | 1.19 | 1.66 | Nr | Med |
| (101) | Ejima et al 2010 | Nigeria | 2010 | 2002-2005 | Cross-sectional | S. h | 5-20 | Mod | Nr | 1.17 | 1.47 | Nr | Med |
| **Ref.** | **Author and year** | **Country** | **Year of publication** | **Study**  **time** | **Study**  **design** | **Species** | **Age**  **range** | **Baseline**  **prevalence** | **Study**  **size** | $\boldsymbol{M:F}$**Prevalence**  **ratios** | $\boldsymbol{M:F}$**Intensity ratios** | **Risk factors identified** | **Ref.** |
| (102) | Oladejo et al 2006 | Nigeria | 2006 | 2001 | Cross-sectional | S. h | <10-13+ | Mod | M=194  F=126 | 0.91 | 0.66 | Nr | Med |
| (103) | Sarda et al 1985 | Tanzania | 1985 | 1984 | Cross-sectional | S. h | 7-19 | Mod | M=1195  F=1223 | 1.57 | Nr | Nr | High |
| (104) | Houmsou et al 2016 | Nigeria | 2016 | Nr | Cross-sectional | S. h | 1-15 | High | M=624  F=541 | 1.67 | Nr | Nr | Med |
| (105) | Ekpo et al 2010 | Nigeria | 2010 | Nr | Observation | S. h | 1-6 | High | M=91  F=76 | 1.03 | 0.96 | Nr | Med |
| (106) | Dabo et al 2011 | Mali | 2011 | Nr | Cross-sectional | S. h | 1-4 | High | M=189  F=149 | 1.03 | 0.88 | Nr | Med |
| (107) | Okoli et al 1999 | Nigeria | 1999 | Nr | Cross-sectional | S. h | 5-17 | Mod | Nr | 2.83 | 176 | Nr | Med |
| (108) | Mafiana et al 2003 | Nigeria | 2003 | Nr | Cross-sectional | S. h | 1-5 | High | M=123  F=86 | 1.03 | Nr | Nr | Med |
| (109) | Noriode et al 2018 | Nigeria | 2011 | Nr | Cross-sectional | S. h | 5-19 | High | M=142  F=109 | 0.91 | Nr | Nr | Med |
| (110) | Ekanem et al 2017 | Nigeria | 2017 | Nr | Cross-sectional | S. h | 5-14 | NA | M=203  F=177 | 0.9 | Nr | Educational level of parents and the proximity to the stream. | Med |
| **Ref.** | **Author and year** | **Country** | **Year of publication** | **Study**  **time** | **Study**  **design** | **Species** | **Age**  **range** | **Baseline**  **prevalence** | **Study**  **size** | $\boldsymbol{M:F}$**Prevalence**  **ratios** | $\boldsymbol{M:F}$**Intensity ratios** | **Risk factors identified** | **Ref.** |
| (111) | Okeke et al 2013 | Nigeria | 2013 | 2012 | Cross-sectional | S. h | 4-15 | Low | M=166  F=157 | 1.08 | 1.15 | Water contact | Med |
| (112) | King et al 2 1988 | Kenya | 1988 | Nr | Cross-sectional | S. h | 0-70 | High | M=288  F=351 | 1.08 | 1.51 | Number of streams visited | High |
| (113) | Adesola et al 2012 | Nigeria | 2012 |  | Cross-sectional | S. h | 3-20 | High | M=252  F=204 | 1.01 | 1.02 | Nr | Med |
| (114) | Abdulkareem et al 2018 | Nigeria | 2018 | 2016 | Cross-sectional | S. h | 4-18 | Mod | M=415  F=309 | 1.20 | 2.33 | Ajase-Ipo – frequency of water contact, source of water supple, unemployment and knowledge level | Med |
| (115) | Atalabi et al 2016 | Nigeria | 2016 | 2015 | Cross-sectional | S. h | 12-25 | Mod | M=405  F=240 | 7.75 | 6.44 | Nr | High |
| (116) | Mutsaka- Makuvaza et al 2019 | Zimbabwe | 2019 | 2016 | Cross-sectional | S. h | 0-5 and 17-49 | Mod | M=291  F=569 | 0.73 | Nr | Nr | High |
| (117) | Salawu et al 2014 | Nigeria | 2014 |  | Cross-sectional | S. h | 1-5 | Low | M=218  F=201 | 0.97 | 1.23 | Water contact activities, farmer fathers | Med |
| (118) | Phillips et al 2018 | Mozambique | 2018 | 2011 | Cross-sectional | S. h | 5-8, 9-12, 20-55 | High | M=11424  F=7538 | 1.07 | Nr | Water source used by, and knowledge level of caregivers | Med |
| **Ref.** | **Author and year** | **Country** | **Year of publication** | **Study**  **time** | **Study**  **design** | **Species** | **Age**  **range** | **Baseline**  **prevalence** | **Study**  **size** | $\boldsymbol{M:F}$**Prevalence**  **ratios** | $\boldsymbol{M:F}$**Intensity ratios** | **Risk factors identified** | **Ref.** |
| (119) | Simoonga et al 2017 | Zambia | 2017 |  | Cross-sectional | S. h | 6-15 | Low | M=1069  F=834 | 1.35 | Nr | Nr | Med |
| (120) | Elom et al 2017 | Nigeria | 2017 | Nr | Cross-sectional | S. h | 4-15 | Mod | M=176  F=226 | 1.16 | Nr | Ezeube  Living on the plateau compared to the valley  Both  Water contact activities and presence of pit latrine | Med |
| (121) | Tukahebwa et al 2013 | Uganda | 2013 | NA | Cross-sectional | S. m | 7-76 | High | M=229  F=217 | 1.28 | 2.79 | Nr | High |
| (122) | Awosolu et al 2020 | Nigeria | 2020 | NA | Cross-sectional | S. h | 3-22 | High | M=362  F=258 | 1.31 | 1.99 | Fishing, number of times people had water contact, duration of time in study area, farming as an occupation or occupation of parent, leisure and domestic use of water. | High |
| (123) | Clennon et al 2004 | Kenya | 2004 | 2000 | Cross-sectional | S.h | 1-92 | High | - | 0.93 | 1.23 | Differences in infection burden between males and females attributed to agricultural and religious practices (e.g., division of labour) | Med |
| **Ref.** | **Author and year** | **Country** | **Year of publication** | **Study**  **time** | **Study**  **design** | **Species** | **Age**  **range** | **Baseline**  **prevalence** | **Study**  **size** | $\boldsymbol{M:F}$**Prevalence**  **ratios** | $\boldsymbol{M:F}$**Intensity ratios** | **Risk factors identified** | **Ref.** |
| (124) | Erko et al 2013 | Ethiopia | 2013 | 2010 | Cross-sectional | S.m | 8-12 | High | M=306  F=314 | 1.16 | Nr | Nr |  |
| (125) | Kinunghi et al 2016 | Tanzania | 2016 | 2011 | Cohort | S.m | 7-8 | High | M=257  F=315 | 0.91 | 0.6 | Nr |  |
| (126) | Olsen et al 2015 | Tanzania | 2015 | 2011 | Cross-sectional | S.m | 7-55 | Moderate | M=16513  F=15352 | 1.09 | 1.09 | Age |  |
| (127) | Kabatereine et al 2004 | Uganda | 2004 | 1998 | Nr | S.m | 5-21  1-90 | Moderate | M=7164  F=6634  M= 5402  F=4427 | School survey 1.07  Community survey  1.29 | Nr | Nr |  |
| (128) | Useh et al | Nigeria | 1999 | 1998 | Nr | S.h |  | Moderate | M=140  F=140 | In school  1.11  Out of school  1.15 | Nr | Nr |  |

**References**

1. Phiri BBW, Ngwira B, Kazembe LN. Analysing risk factors of co-occurrence of schistosomiasis haematobium and hookworm using bivariate regression models: Case study of Chikwawa, Malawi. Parasite Epidemiology and Control. 2016;1(2):149-58.

2. Rasoamanamihaja CF, Rahetilahy AM, Ranjatoarivony B, Dhanani N, Andriamaro L, Andrianarisoa SH, et al. Baseline prevalence and intensity of schistosomiasis at sentinel sites in Madagascar: Informing a national control strategy. Parasit Vectors. 2016;9:50.

3. Chisango TJ, Bongiwe N, Vengesai A, Nhidza AF, Sibanda EP, Zhou D, et al. Benefits of annual chemotherapeutic control of schistosomaisis on the development of protective immunity. . BMC Infectious Diseases. 2019;19(219).

4. Elmorshedy H, Bergquist R, El-Ela NE, Eassa SM, Elsakka EE, Barakat R. Can human schistosomiasis mansoni control be sustained in high-risk transmission foci in Egypt? Parasit Vectors. 2015;8:372.

5. King CH, Lombardi G, Lombardi C, Greenblatt R, Hodder S, Kinyanjui H, et al. Chemotherapy-based control of schistosomiasis haematobia. I. Metrifonate versus praziquantel in control of intensity and prevalence of infection. American Journal of Tropical Medicine and Hygiene. 1988;39(3):295-305.

6. Abebe N, Erko B, Girmay M, Berhe N. Clinico-epidemiology study of Schistosoma mansoni in Waja-Timuga, District of Alamata, northern Ethiopia. Parasites & Vectors. 2014;7(158).

7. Mohammed J, Weldegebreal F, Tekelmariam Z, Mitiku H. Clinico-epidmeiology, malacoloy and community awareness of Schistosoma mansoni in Haradenaba and Dertoramis kebeles in Debeno district, eastern Ethiopia. Sage Open Medicine. 2018;6:1-11.

8. Mazigo HD, Dunne DW, Wilson S, Kinung'hi SM, Pinot de Moira A, Jones FM, et al. Co-infection with Schistosoma mansoni and Human Immunodeficiency Virus-1(HIV-1) among residents of fishing villages of north-western Tanzania. Parasites & Vectors. 2014;7(587).

9. Ogbonna CC, Dori GU, Nweze EI, Muoneke G, Nwankwo IE, Akputa N. Comparative analysis of urinary schistosomiasis among primary school children and rural farmers in Obollo–Eke, Enugu State, Nigeria: Implications for control. Asian Pacific Journal of Tropical Medicine. 2012;5(10):796-802.

10. Russell HJ, Penney JMS, Linder C, Joekes EC, Bustinduy AL, Stothard JR, et al. A cross-sectional study of periportal fibrosis and Schistosoma mansoni infection among school-aged children in a hard-to-reach area of Madagascar. Transactions of the Royal Society of Tropical Medicine and Hygiene. 2020;114(4):315-22.

11. Morenikeji O, Quazim J, Omoregie C, Hassan A, Nwuba R, Anumudu C, et al. A cross-sectional study on urogenital schistosomiasis in children; haematuria and proteinuria as diagnostic indicators in an endemic rural area of Nigeria. Afr Health Sci. 2014;14(2):390-6.

12. Hodges M, Koroma MM, Balde MS, Turay H, Fofanah I, Divall MJ, et al. Current status of schistosomiasis and soil-transmitted helminthiasis in Beyla and Macenta Prefectures, Forest Guinea. Trans R Soc Trop Med Hyg. 2011;105(11):672-4.

13. Bourke CD, Nausch N, Rujeni N, Appleby LJ, Trottein F, Midzi N, et al. Cytokine responses to the anti-schistosome vaccine candidate antigen glutathione-S-transferase vary with host age and are boosted by praziquantel treatment. PLoS Negl Trop Dis. 2014;8(5):e2846.

14. Kahama AI, Nibbeling HAM, Van Zeyl RJM, Vennervald BJ, Ouma JH, Deelder AM. Detection and quantification of soluble egg antigen in urine of Schistosoma haematobium infected children from Kenya. American Journal of Tropical Medicine and Hygiene. 1998;59:769-74.

15. Midzi N, Mduluza T, Chimbari MJ, Tshuma C, Charimari L, Mhlanga G, et al. Distribution of schistosomiasis and soil transmitted helminthiasis in Zimbabwe: towards a national plan of action for control and elimination. PLoS Negl Trop Dis. 2014;8(8):e3014.

16. Joseph SO, Abdulkareem BO, Samuel UU. Distribution Pattern of Human Urinary Schistosomiasis in Kwara State, Nigeria. American Journal of Infectious Diseases. 2017;13(4):38-44.

17. Ebai CB, Kimbi HK, Sumbele IUN, Yunga JE, Lehman LG. Efficacy and safety of praziquantel against Schistosoma haematobium in the Ikata-Likoko area of southwest Cameroon. Trop Med Health. 2017;45:30.

18. Raso G, N’Goran EK, Toty A, Luginbühl A, Adjoua CA, Tian-Bi NT, et al. Efficacy and side effects of praziquantel against Schistosoma mansoni in a community of western Côte d’Ivoire. Transactions of the Royal Society of Tropical Medicine and Hygiene. 2004;98(1):18-27.

19. Erko B, Degarege A, Tadesse K, Mathiwos A, Legesse M. Efficacy and side effects of praziquantel in the treatment of Schistosomiasis mansoni in schoolchildren in Shesha Kekele Elementary School, Wondo Genet, Southern Ethiopia. Asian Pacific Journal of Tropical Biomedicine. 2012;2(3):235-9.

20. N'Goran EK, Gnaka HN, Tanner M, Utzinger J. Efficacy and side-effects of two praziquantel treatments against Schistosoma haematobium infection, among schoolchildren from Cote d'Ivoire. Ann Trop Med Parasitol. 2003;97(1):37-51.

21. Senghor B, Diaw OT, Doucoure S, Sylla SN, Seye M, Talla I, et al. Efficacy of praziquantel against urinary schistosomiasis and reinfection in Senegalese school children where there is a single well-defined transmission period. Parasit Vectors. 2015;8:362.

22. Haggag AA, Rabiee A, Abd Elaziz KM, Gabrielli AF, Abdelhai R, Hashish A, et al. Elimination of schistosomiasis haematobia as a public health problem in five governorates in Upper Egypt. Acta Trop. 2018;188:9-15.

23. Ntonifor HN, Mbunker GN, Ndaleh NW. Epidemiological Survey of Urinary Schistosomiasis in Some Primary Schools in a New Focus Behind Mount Camaroon, South WEst Region, Cameroon. . East African Medical Journal. 2012;89.

24. Lee YH, Lee JS, Jeoung HG, Kwon IS, Mohamed A, Hong ST. Epidemiological Survey on Schistosomiasis and Intestinal Helminthiasis among Village Residents of the Rural River Basin Area in White Nile State, Sudan. Korean J Parasitol. 2019;57(2):135-44.

25. Clercq DD, Vercruysse J, Picqut M, Shaw DJ, Diop M, Ly A, et al. The epidemiology of a recent focus of mixed Schistosoma haematobium and Schistosoma mansoni infections around the “Lac de Guiers” in the Senegal River Basin, Senegal Tropical Medicine and International Health. 1999;4:544-50.

26. Tefera A, Belay T, Bajiro M. Epidemiology of Schistosoma mansoni infection and associated risk factors among school children attending primary schools nearby rivers in Jimma town, an urban setting, Southwest Ethiopia. PLoS One. 2020;15(2):e0228007.

27. Stelma FF, Talla I, Polman K, Niang M, Sturrock RF, Deelder AM, et al. Epidemiology of Schistosoma mansoni infection in a recently exposed community in Northern Senegal. American Journal of Tropical Medicine and Hygiene. 1993;49(6):701-6.

28. Afifi A, Abdel-Aziz AA, Sulieman Y, Pengsakul T. Epidemiology of schistosomiasis among villagers of the New Halfa Agricultural Scheme, Sudan. Iranian Journal of Parasitology. 2015;11:110-5.

29. Abdel-Wahab MF, Esmat G, Ramzy I, Narooz S, Medhat Ed, Ibrahim M, et al. The Epidemiology of schistosomiasis in Egypt: Fayoum Governorate. American Journal of Tropical Medicine and Hygiene. 2000;62(S):55-64.

30. El-Hawey AM, Amr MM, Abdel-Rahman AH, El-Ibiary SA, Agina AM, Abdel-Hafez MA, et al. The epidemiology of schistosomiasis in Egypt: Gharbia Governorate. American Journal of Tropical Medicine and Hygiene. 2000;62(S):42-8.

31. Nooman ZM, Hasan AH, Waheeb Y, Mishriky AM, Ragheb M, Abu-Saif AN, et al. The epidemiology of schistosomiasis in Egypt: Ismailia Governorate. American Journal of Tropical Medicine and Hygiene. 2000;62(S):35-41.

32. Abdel-Wahab MF, Esmat G, Medhat E, Narooz S, Ramzy I, El-Boraey Y, et al. The epidemiology of schistosomiasis in Egypt: Menofia Governorate. American Journal of Tropical Medicine and Hygiene. 2000;62(S):28-34.

33. Barakat R, Farghaly A, El Masry AG, El-Sayed M, Hussein MH. The epidemiology of schistosomiasis in Egypt: Patterns of Schistosoma mansoni infection and morbidity in Kafr El-Sheikh. American Journal of Tropical Medicine and Hygiene. 2000;62(S):21-7.

34. El-Khoby T, Galal N, Fenwick A, Barakat R, El-Hawey AM, Nooman ZM, et al. The epidmemiology of schistosomiasis in Egypt: Summary findings in nine governorates. American Journal of Tropical Medicine and Hygiene. 2000;62(S):88-99.

35. Birrie H, Abebe F, Gundersen SG, Medhin G, Berhe N, Gemetchu T. Epidemiology of schistosomiasis mansoni in three endemic communities in north-east Ethiopia: baseline characteristics before endod based intervention. Ethiopian Medical Journal. 1998;36:101-11.

36. Sulieman Y, Eltayeb RE, Pengsakul T, Afifi A, Zakaria MA. Epidemiology of urinary schistosomiasis among school children in the alsaial Alsagair village, River nile state, Sudan. Iranian Journal of Parasitology. 2017;12(2):284-91.

37. Ibrahim AM, Ibrahim ME. Evaluation of microscopical and serological techniques in the diagnosis of Schistosoma mansoni infection at Sennar State, Central Sudan. Asian Pacific Journal of Tropical Disease. 2014;4(1):8-13.

38. Ugbomoiko US, Ofoezie IE, Okoye IC, Heukelbach J. Factors associated with urinary schistosomiasis in two peri-urban communities in south-western Nigeria. Ann Trop Med Parasitol. 2010;104(5):409-19.

39. Satayathum SA, Muchiri EM, Ouma JH, Whalen CC, King CH. Factors affecting infection or reinfection with Schistosoma haematobium in coastal Kenya: Survival analysis during a nine-year, school-based treatment program. American Journal of Tropical Medicine and Hygiene. 2012;75:83-92.

40. Opara KN, Udoidung NI, Ukpong IG. Genitourinary schistosomiasis among pre-primary schoolchildren in a rural community within the Cross River Basin, Nigeria. J Helminthol. 2007;81(4):393-7.

41. El Scheich T, Hofer L, Kaatano G, Foya J, Odhiambo D, Igogote J, et al. Hepatosplenic morbidity due to Schistosoma mansoni in schoolchildren on Ukerewe Island, Tanzania. Parasitology Research. 2012;110(6):2515-20.

42. Ahmed AM, El Tash LA, Mohamed EY, Adam I. High levels of Schistosoma mansoni infections among schoolchildren in central Sudan one year after treatment with praziquantel. Journal of Helminthology. 2012;86(2):228-32.

43. Mutengo MM, Mwansa JC, Mduluza T, Sianongo S, Chipeta J. High Schistosoma mansoni disease burden in a rural district of western Zambia. Am J Trop Med Hyg. 2014;91(5):965-72.

44. Ito EE. Hyper-endemicity of urinary schistosomiasis in two communities in lower Niger Delta, southern Nigeria. Nigerian Journal of Parasitology. 2019;40(1).

45. Hodges M, Dada N, Wamsley A, Paye J, Nyorkor E, Sonnie M, et al. Improved mapping strategy to better inform policy on the control of schistosomiasis and soil-transmitted helminthiasis in Sierra Leone. Parasit Vectors. 2011;4:97.

46. Augusto G, Magnussen P, Kristensen TK, Appleton CC, Vennervald BJ. The influence of transmission season on parasitological cure rates and intensity of infection after praziquantel treatment of Schistosoma haematobium-infected schoolchildren in Mozambique. Parasitology. 2009;136(13):1771-9.

47. Nalugwa A, Olsen A, Tukahebwa ME, Nuwaha F. Intestinal schistosomiasis among preschool children along the shores of Lake Victoria in Uganda. Acta Tropica. 2015;142:115-21.

48. Mugono M, Konje E, Kuhn S, Mpogoro FJ, Morona D, Mazigo HD. Intestinal schistosomiasis and geohelminths of Ukara Island, North-Western Tanzania: prevalence, intensity of infection and associated risk factors among school children. Parasit Vectors. 2014;7:612.

49. Mueller A, Fuss A, Ziegler U, Kaatano GM, Mazigo HD. Intestinal schistosomiasis of Ijinga Island, north-western Tanzania: prevalence, intensity of infection, hepatosplenic morbidities and their associated factors. BMC Infect Dis. 2019;19(1):832.

50. Emejulu AC, Alabaronye FF, Ezenwaji HM, Okafor FC. Investigation into the prevalence of urinary schistosomiasis in the Agulu Lake area of Anambra State, Nigeria. Journal of Helminthology. 1994;68:119-23.

51. Soares Magalhaes RJ, Biritwum NK, Gyapong JO, Brooker S, Zhang Y, Blair L, et al. Mapping helminth co-infection and co-intensity: geostatistical prediction in ghana. PLoS Negl Trop Dis. 2011;5(6):e1200.

52. Mwandawiro CS, Nikolay B, Kihara JH, Ozier O, Mukoko DA, Mwanje MT, et al. Monitoring and evaluating the impact of national school-based deworming in Kenya: study design and baseline results. Parasites & Vectors. 2013;6.

53. Raso G, Luginbuhl A, Adjoua CA, Tian-Bi NT, Silue KD, Matthys B, et al. Multiple parasite infections and their relationship to self-reported morbidity in a community of rural Cote d'Ivoire. Int J Epidemiol. 2004;33(5):1092-102.

54. Moyou-Somo R, Jouemeni LE, Ndjamen B, Ngogang J, Dongla R, Longag-Tchatchouang V, et al. A new focus of Schistosoma mansoni in Yoro Village, Mbam and Inoubou Division, Cameroon American Journal of Tropical Medicine and Hygiene. 2003;69(1):74-7.

55. Stecher CW, Madsen H, Wilson S, Sacko M, Wejse C, Keita AD, et al. Organomegaly in Mali before and after praziquantel treatment. A possible association with Schistosoma haematobium. Heliyon. 2017;3(11):e00440.

56. Nwabueze AA, Opara KN. Outbreak of Urinary Schistosomiasis among School Children in Riverine Communities of Delta State, Nigeria: Impact of Road and Bridge Construction. Journal of Medical Sciences(Faisalabad). 2007;7(4):572-8.

57. Zhang Y, Koukounari A, Kabatereine N, Fleming F, Kazibwe F, Tukahebwa E, et al. Parasitological impact of 2-year preventive chemotherapy on schistosomiasis and soil-transmitted helminthiasis in Uganda. BMC Med. 2007;5:27.

58. Randall AE, Perez MA, Floyd S, Black GF, Crampin AC, Ngwira B, et al. Patterns of helminth infection and relationship to BCG vaccination in Karonga District, northern Malawi. Transactions of the Royal Society of Tropical Medicine and Hygiene. 2002;96(1):29-33.

59. Saathoff E, Olsen A, Magnussen P, Kvalsvig JD, Becker W, Appleton CC. Patterns of Schistosoma haematobium infection, impact of praziquantel treatment and re-infection after treatment in a cohort of schoolchildren from rural KwaZulu-Natal/South Africa. BMC Infect Dis. 2004;4:40.

60. Mnkugwe RH, Minzi OS, Kinung'hi SM, Kamuhabwa AA, Aklillu E. Prevalence and correlates of intestinal schistosomiasis infection among school-aged children in North-Western Tanzania. PLoS One. 2020;15(2):e0228770.

61. Atalabi TE, Lawal U, Ipinlaye SJ. Prevalence and intensity of genito-urinary schistosomiasis and associated risk factors among junior high school students in two local government areas around Zobe Dam in Katsina State, Nigeria. Parasit Vectors. 2016;9(1):388.

62. Ndokeji S, Mazigo HD, Temu M, Kishamawe C, Malenganisho W, Todd J, et al. Prevalence and intensity of Schistosoma mansoniand hookworm infections among pre-school and school-aged children in Llemela District, north-western Tanzania. Tanzania Journal of Health Research. 2016;18(2).

63. Mazigo HD, Kirway L, Ambrose EA. Prevalence and intensity of Schistosoma mansoni infection in pediatric populations on antiretroviral therapy in north-western Tanzania: a cross-sectional study. BMJ Open. 2019;9(7):e029749.

64. Bajiro M, Dana D, Levecke B. Prevalence and intensity of Schistosoma mansoni infections among schoolchildren attending primary schools in an urban setting in Southwest, Ethiopia. BMC Res Notes. 2017;10(1):677.

65. Senghor B, Diallo A, Sylla SN, Doucoure S, Ndiath MO, Gaayeb L, et al. Prevalence and intensity of urinary schistosomiasis among school children in the district of Niakhar, region of Fatick, Senegal. Parasites & Vectors. 2014;7(5).

66. Ndamukong KJ, Ayuk MA, Dinga JS, Akenji TN, Ndiforchu VA, Titanji VP. Prevalence and intensity of urinary schistosomiasis in primary school children of the Kotto Barombi Health Area, Cameroon. East African Medical Journal. 2001;78(6):287-9.

67. Woldegerima E, Bayih AG, Tegegne Y, Aemero M, Jejaw Zeleke A. Prevalence and Reinfection Rates of Schistosoma mansoni and Praziquantel Efficacy against the Parasite among Primary School Children in Sanja Town, Northwest Ethiopia. J Parasitol Res. 2019;2019:3697216.

68. Ndassi VD, Anchang-Kimbi JK, Sumbele IUN, Wepnje GB, Kimbi HK. Prevalence and Risk Factors Associated with S. haematobium Egg Excretion during the Dry Season, Six Months following Mass Distribution of Praziquantel (PZQ) in 2017 in the Bafia Health Area, South West Region Cameroon: A Cross-Sectional Study. J Parasitol Res. 2019;2019:4397263.

69. Sacolo-Gwebu H, Chimbari M, Kalinda C. Prevalence and risk factors of schistosomiasis and soil-transmitted helminthiases among preschool aged children (1-5 years) in rural KwaZulu-Natal, South Africa: a cross-sectional study. Infect Dis Poverty. 2019;8(1):47.

70. Ivoke N, Ivoke ON, Nwani CD, Ekeh FN, Asogwa CN, Atama CI, et al. Prevalence and transmission dynamics of Schistosoma haematobium infection in a rural community of south- western Ebonyi State, Nigeria. Tropical Biomedicine. 2014;31(1):77-88.

71. Gebreyohanns A, Legese MH, Wolde M, Leta G, Tasew G. Prevalence of intestinal parasites versus knowledge, attitude and practices (KAPs) with special emphasis to Schistosoma mansoni among individuals who have river water contact in Addiremets town, Western Tigray, Ethiopia. PLoS One. 2018;13(9):e0204259.

72. Degarege A, Mekonnen Z, Levecke B, Legesse M, Negash Y, Vercruysse J, et al. Prevalence of Schistosoma haematobium Infection among School-Age Children in Afar Area, Northeastern Ethiopia. PLoS One. 2015;10(8):e0133142.

73. Bajiro M, Dana D, Ayana M, Emana D, Mekonnen Z, Zawdie B, et al. Prevalence of Schistosoma mansoni infection and the therapeutic efficacy of praziquantel among school children in Manna District, Jimma Zone, southwest Ethiopia. Parasit Vectors. 2016;9(1):560.

74. Khonde Kumbu R, Mbanzulu Makola K, Bin L. Prevalence of Schistosoma mansoni Infection in Four Health Areas of Kisantu Health Zone, Democratic Republic of the Congo. Adv Med. 2016;2016:6596095.

75. Nute AW, Endeshaw T, Stewart AEP, Sata E, Bayissasse B, Zerihun M, et al. Prevalence of soil-transmitted helminths and Schistosoma mansoni among a population-based sample of school-age children in Amhara region, Ethiopia. Parasit Vectors. 2018;11(1):431.

76. Oluwole AS, Adeniran AA, Mogaji HO, Olabinke DB, Abe EM, Bankole SO, et al. Prevalence, intensity and spatial co-distribution of schistosomiasis and soil transmitted helminths infections in Ogun state, Nigeria. Parasitology Open. 2018;4.

77. Amuta EU, Houmsou RS. Prevalence, intensity of infection and risk factors of urinary schistosomiasis in pre-school and school aged children in Guma Local Government Area, Nigeria. Asian Pacific Journal of Tropical Medicine. 2014;7(1):34-9.

78. Ismail HAHA, Hong ST, Babiker ATEB, Hassan RMAE, Sulaiman MAZ, Jeong H-G, et al. Prevalence, risk factors, and clinical manifestations of schistosomiasis among school children in the White Nile River basin, Sudan. Parasites & Vectors. 2014;7(478).

79. Naus CWA, Booth M, Jones FM, Kembijumbi J, Vennervald BJ, Kariuki CH, et al. The relationship between age, sex, egg-count and specific antibody responses against Schistosoma mansoni antigens in a Ugandan fishing community. Tropical Medicine and International Health. 2003;8(6):561-8.

80. Clercq DD, Sacko M, Behnke J, Gilbert F, Vercruysse J. The relationship between Schistosoma haematobium infection and school performance and attendance in Bamako, Mali. Annals of Tropical Medicine and Parasitology. 1998;92(8):851-8.

81. Kimani BW, Mbugua AK, Kihara JH, Ng'ang'a M, Njomo DW. Safety, efficacy and acceptability of praziquantel in the treatment of Schistosoma haematobium in pre-school children of Kwale County, Kenya. PLoS Negl Trop Dis. 2018;12(10):e0006852.

82. Abdel-Wahab MF, Esmat G, Ramzy I, Fouad R, Abdel-Rahman AH, Yosery A, et al. Schistosoma haematobium infection in Egyptian schoolchildren: Demonstration of both hepatic and urinary tract morbidity by ultrasonography. Transactions of the Royal Society of Tropical Medicine and Hygiene. 1992;86:406-9.

83. Elias E, Daffala A, Lassen JM, Madsen H, Christensen NØ. Schistosoma haematobium infection patterns in the Rahad Irrigation Scheme, Sudan. Acta Tropica. 1994;58:115-25.

84. Ahmed AM, Abbas H, Mansour FA, Gasim GI, Adam I. Schistosoma haematobium infections among schoolchildren in central Sudan one year after treatment with praziquantel. Parasites & Vectors. 2012;108.

85. Ekpo UF, Alabi OM, Oluwole AS, Sam-Wobo SO. Schistosoma haematobium infections in preschool children from two rural communities in Ijebu East, south-western Nigeria. J Helminthol. 2012;86(3):323-8.

86. Bello AB, Edungbola LD. Schistosoma haematobium: a neglected common parasitic disease of childhood in Nigeria. Incidence and intensity of infection. Acta Paediatrica. 1992;81(8):601-4.

87. Ruganuza DM, Mazigo HD, Waihenya R, Morona D, Mkoji GM. Schistosoma mansoni among pre-school children in Musozi village, Ukerewe Island, North-Western-Tanzania: prevalence and associated risk factors. Parasit Vectors. 2015;8:377.

88. Alemu A, Tegegne Y, Damte D, Melku M. Schistosoma mansoni and soil-transmitted helminths among preschool-aged children in Chuahit, Dembia district, Northwest Ethiopia: prevalence, intensity of infection and associated risk factors. BMC Public Health. 2016;16:422.

89. Mazigo HD, Nuwaha F, Dunne DW, Kaatano GM, Angelo T, Kepha S, et al. Schistosoma mansoni Infection and Its Related Morbidity among Adults Living in Selected Villages of Mara Region, North-Western Tanzania: A Cross-Sectional Exploratory Study. Korean J Parasitol. 2017;55(5):533-40.

90. Alemayehu B, Tomass Z. Schistosoma mansoni infection prevalence and associated risk factors among schoolchildren in Demba Girara, Damot Woide District of Wolaita Zone, Southern Ethiopia. Asian Pac J Trop Med. 2015;8(6):457-63.

91. Nalugwa A, Nuwaha F, Tukahebwa EM, Olsen A. Schistosoma mansoni-Associated Morbidity among Preschool-Aged Children along the Shores of Lake Victoria in Uganda. Trop Med Infect Dis. 2017;2(4).

92. Bah YM, Paye J, Bah MS, Conteh A, Saffa S, Tia A, et al. Schistosomiasis in School Age Children in Sierra Leone After 6 Years of Mass Drug Administration With Praziquantel. Front Public Health. 2019;7:1.

93. Terefe A, Chimelis T, Mengistu M, Hailu AD, Erko B. Schistosomiasis mansoni and soil-transmitted helminthiasis in Bushulo village, southern Ethiopia. Ethiopian Journal of Health Development. 2011;25(1).

94. Abdel-Wahab MF, Strickland GT, El-Sahly A, Ahmed L, Zakaria S, El Kady N, et al. Schistosomiasis mansoni in an Egyptian village in the Nile delta. American Journal of Tropical Medicine and Hygiene. 1980;29(5):868-74.

95. John R, Ezekiel M, Philbert C, Andrew A. Schistosomiasis transmission at high altitude crater lakes in western Uganda. BMC Infect Dis. 2008;8:110.

96. Landoure A, Dembele R, Goita S, Kane M, Tuinsma M, Sacko M, et al. Significantly reduced intensity of infection but persistent prevalence of schistosomiasis in a highly endemic region in Mali after repeated treatment. PLoS Neglected Tropical Diseases. 2012;6(7):e1774.

97. Aemero M, Berhe N, Erko B. Status of Schistosoma mansoni prevalence and intensity of infection in geographically apart endemic localities of Ethiopia: a comparison. Ethiopian Journal of Health Science. 2014;24(3):189-94.

98. Vang Johansen M, Simonsen PE, Butterworth AE, Ouma JH, Mbugua GG, Sturrock RF, et al. A survey for Schistosoma mansoni induced kidney disease in children in an endemic area of Machakos District, Kenya. Acta Tropica. 1994;50:21-18.

99. Mohamed-Ali Q, Elwali N-EMA, Abdelhameed AA, Mergani A, Rahoud S, Elagib KE, et al. Susceptibility to Periportal (Symmers) Fibrosis in Human Schistosoma mansoni Infections: Evidence That Intensity and Duration of Infection, Gender,

and Inherited Factors Are Critical in Disease Progression. The Journal of Infectious Diseases. 1999;180:1298-306.

100. Toure S, Zhang Y, Bosque-Oliva E, Ky C, Ouedraogo A, Koukounari A, et al. Two-year impact of single praziquantel treatment on infection in the national control programme on schistosomiasis in Burkina Faso. Bull World Health Organ. 2008;86(10):780-7, A.

101. Ejima IAA, Odaibo A. Urinary schistomiasis in the Niger-Benue basin of Kogi state, Nigeria. International Journal of Tropical Medicine. 2010;5(3):73-80.

102. Oladejo SO, Ofoezie IE. Unabated schistosomiasis transmission in Erinle River Dam, Osun State, Nigeria: evidence of neglect of environmental effects of development projects. Trop Med Int Health. 2006;11(6):843-50.

103. Sarda RK, Simonsen PE, Mahikwano LF. Urban transmission of urinary schistosomiasis in Dar es Salaam, Tanzania. Acta Tropica. 1985;42(1):71-8.

104. Houmsou RS, Agere H, Wama BE, Bingbeng JB, Amuta EU, Kela SL. Urinary Schistosomiasis among Children in Murbai and Surbai Communities of Ardo-Kola Local Government Area, Taraba State, Nigeria. J Trop Med. 2016;2016:9831265.

105. Ekpo UF, Laja-Deile A, Oluwole AS, Sam-Wobo SO, Mafiana CF. Urinary schistosomiasis among preschool children in a rural community near Abeokuta, Nigeria. Parasites & Vectors. 2010;3.

106. Dabo A, Badawi HM, Bary B, Doumbo OK. Urinary schistosomiasis among preschool-aged children in Sahelian rural communities in Mali. Parasit Vectors. 2011;4:21.

107. Okoli EI, Odaibo A. Urinary schistosomiasis among schoolchildren in Ibadan, an urban community in south-western Nigeria. Tropical Medicine and International Health. 1999;4:308-15.

108. Mafiana CF, Ekpo UF, Ojo DA. Urinary schistosomiasis in preschool children in settlements around Oyan Reservoir in Ogun State, Nigeria: Implications for control. Tropical Medicine and International Health. 2003;8(1):78-82.

109. Noriode RM, Idowu ET, Otubanjo OA, Mafe MA. Urinary schistosomiasis in school aged children of two rural endemic communities in Edo State, Nigeria. J Infect Public Health. 2018;11(3):384-8.

110. Ekanem EE, Akapan FM, Eyong ME. Urinary schistosomiasis in school children of a southern nigerian community 8 years after the provision of potable water. Niger Postgrad Med J. 2017;24(4):201-4.

111. Okeke OC, Ubachukwu PO. Urinary schistosomiasis in urban and semi-urban communities in South-Eastern Nigeria. Iranian Journal of Parasitology. 2013;8(3):467-73.

112. King C, Keating CE, Muruka JF, Ouma JH, Houser H, Siongok TKA, et al. Urinary tract morbidity in schistosomiasis haematobia: Associations with age and intensity of infection in an endemic area of Coast Provine, Kenya. American Journal of Tropical Medicine and Hygiene. 1988;39(4):361-98.

113. Adesola H, Uduak N, Olajumoke M, Roseangela N, Chiaka A, Sunday A, et al. Urine Turbidity and Microhaematuria as Rapid Assessment Indicators for Schistosoma haematobium Infection among School Children in Endemic Areas. American Journal of Infectious Diseases. 2012;8(1):60-4.

114. Abdulkareem BO, Habeeb KO, Kazeem A, Adam AO, Samuel UU. Urogenital Schistosomiasis among Schoolchildren and the Associated Risk Factors in Selected Rural Communities of Kwara State, Nigeria. Journal of Tropical Medicine. 2018;2018:6913918.

115. Atalabi TE, Lawal U, Akinluyi FO. Urogenital schistosomiasis and associated determinant factors among senior high school students in the Dutsin-Ma and Safana Local Government Areas of Katsina State, Nigeria. Infect Dis Poverty. 2016;5(1):69.

116. Mutsaka-Makuvaza MJ, Matsena-Zingoni Z, Katsidzira A, Tshuma C, Chin'ombe N, Zhou XN, et al. Urogenital schistosomiasis and risk factors of infection in mothers and preschool children in an endemic district in Zimbabwe. Parasit Vectors. 2019;12(1):427.

117. Salawu OT, Odaibo AB. Urogenital schistosomiasis and urological assessment of hematuria in preschool-aged children in rural communities of Nigeria. J Pediatr Urol. 2014;10(1):88-93.

118. Phillips AE, Gazzinelli-Guimaraes PH, Aurelio HO, Dhanani N, Ferro J, Nala R, et al. Urogenital schistosomiasis in Cabo Delgado, northern Mozambique: baseline findings from the SCORE study. Parasit Vectors. 2018;11(1):30.

119. Simoonga C, Kazembe LN. Using the hierarchical ordinal regression model to analyse the intensity of urinary schistosomiasis infection in school children in Lusaka Province, Zambia. Infect Dis Poverty. 2017;6(1):43.

120. Elom JE, Odikamnoro OO, Nnachi AU, Ikeh I, Nkwuda JO. Variability of Urine Parameters in Children Infected with Schistosoma Haematobium in Ukawu Community, Onicha Local Government Area, Ebonyi State, Nigeria. African Journal of Infectious Diseases 2017;11(2):10-6.

121. Tukahebwa EM, Magnussen P, Madsen H, Kabatereine NB, Nuwaha F, Wilson S, et al. A very high infection intensity of Schistosoma mansoni in a Ugandan Lake Victoria Fishing Community is required for association with highly prevalent organ related morbidity. PLoS Negl Trop Dis. 2013;7(7):e2268.

122. Awosolu OB, Shariman YZ, Haziqah MTF, Olusi TA. Will Nigerians Win the War Against Urinary Schistosomiasis? Prevalence, Intensity, Risk Factors and Knowledge Assessment among Some Rural Communities in Southwestern Nigeria. Pathogens. 2020;9(2).

123. Clennon JA, King CH, Muchiri EM, Kariuki HC, Ouma JH, Mungai P, et al. Spatial patterns of urinary schistosomiasis infection in a highly endemic area of coastal Kenya. The American journal of tropical medicine and hygiene. 2004;70(4):443-8.

124. Erko B, Medhin G, Teklehaymanot T, Degarege A, Legesse M. Evaluation of urine‐circulating cathodic antigen (Urine‐CCA) cassette test for the detection of Schistosoma mansoni infection in areas of moderate prevalence in Ethiopia. Tropical Medicine & International Health. 2013;18(8):1029-35.

125. Kinung’hi S, Magnussen P, Kaatano G, Olsen A. Infection with Schistosoma mansoni has an effect on quality of life, but not on physical fitness in schoolchildren in Mwanza region, north-western Tanzania: a cross-sectional study. PLoS neglected tropical diseases. 2016;10(12):e0005257.

126. Olsen A, Kinung'hi S, Magnussen P. Schistosoma mansoni infection along the coast of Lake Victoria in Mwanza region, Tanzania. The American journal of tropical medicine and hygiene. 2015;92(6):1240.

127. Kabatereine NB, Brooker S, Tukahebwa EM, Kazibwe F, Onapa AW. Epidemiology and geography of Schistosoma mansoni in Uganda: implications for planning control. Trop Med Int Health. 2004;9(3):372-80.

128. Useh MF, Ejezie GC. School-based schistosomiasis control programmes: a comparative study on the prevalence and intensity of urinary schistosomiasis among Nigerian school-age children in and out of school. Trans R Soc Trop Med Hyg. 1999;93(4):387-91.
