# Supplementary material for: Gender-related differences in prevalence, intensity and associated risk factors of Schistosoma infections in Africa: A systematic review and meta-analysis
Source: PLoS Negl Trop Dis. 2021 Nov 17;15(11):e0009083. doi: 10.1371/journal.pntd.0009083 (PMC8635327; doi:10.1371/journal.pntd.0009083)
Supplement: S3 Table — DF = degrees of freedom, * depicts p-value < 0.05. (DOCX) [file pntd.0009083.s003.docx]

**S3 Table**. Results of univariate meta-regression analysis showing the effect of age (lower and upper age limit of included studies), baseline prevalence and sample size on the $M:F$ prevalence of infection ratio of *S. haematobium*. DF = degrees of freedom, ** depicts p-value < 0.05*.

| Moderator | N | Omnibus test ($Q_{M})$ | DF | p-value | Amount of heterogeneity accounted for $\left( R^{2} \right)$ |
| --- | --- | --- | --- | --- | --- |
| Baseline prevalence | 71 | 9.8681 | 1 | 0.0017* | 17.51% |
| Age (lower age limit) | 64 | 11.6687 | 1 | 0.0006* | 18.96% |
| Age (upper age limit) | 64 | 0.0284 | 1 | 0.8662 | 0.00% |
| Sample size | 74 | 6.2835 | 1 | 0.0122* | 11.79% |
