# Supplementary material for: Gender-related differences in prevalence, intensity and associated risk factors of Schistosoma infections in Africa: A systematic review and meta-analysis
Source: PLoS Negl Trop Dis. 2021 Nov 17;15(11):e0009083. doi: 10.1371/journal.pntd.0009083 (PMC8635327; doi:10.1371/journal.pntd.0009083)
Supplement: S4 Table — DF = degrees of freedom, * depicts p-value < 0.05. (DOCX) [file pntd.0009083.s004.docx]

**S4 Table**. Results of univariate meta-regression analysis showing the effect of age (lower and upper age limit of included studies), baseline prevalence and sample size on the $M:F$ prevalence of infection ratio of *S. mansoni*. DF = degrees of freedom, ** depicts p-value < 0.05*.

| Moderator | N | Omnibus test ($Q_{M})$ | DF | p-value | Amount of heterogeneity accounted for $\left( R^{2} \right)$ |
| --- | --- | --- | --- | --- | --- |
| Baseline prevalence | 66 | 7.6447 | 1 | 0.0057* | 10.50% |
| Age (lower age limit) | 59 | 0.4226 | 1 | 0.5156 | 0.00% |
| Age (upper age limit) | 58 | 1.4369 | 1 | 0.2306 | 3.75% |
| Sample size | 65 | 1.1073 | 1 | 0.2927 | 0.99% |
