# Supplementary material for: Gender-related differences in prevalence, intensity and associated risk factors of Schistosoma infections in Africa: A systematic review and meta-analysis
Source: PLoS Negl Trop Dis. 2021 Nov 17;15(11):e0009083. doi: 10.1371/journal.pntd.0009083 (PMC8635327; doi:10.1371/journal.pntd.0009083)
Supplement: S1 Text — (DOCX) [file pntd.0009083.s005.docx]

## **S1 Text: Search strategy**

(("Schistosomiasis" or "Schistosoma mansoni" or "urogenital schistosome" or "Schistosoma haematobium" or "Urinary Schistosomiasis" or "Intestinal Schistosomiasis" or "bilharzia" or "bilharziasis") and ("Prevalence" or "Burden") and ("infection intensity" or "intensity of infection"))
