# Supplementary material for: Gender-related differences in prevalence, intensity and associated risk factors of Schistosoma infections in Africa: A systematic review and meta-analysis
Source: PLoS Negl Trop Dis. 2021 Nov 17;15(11):e0009083. doi: 10.1371/journal.pntd.0009083 (PMC8635327; doi:10.1371/journal.pntd.0009083)
Supplement: S2 Text — (DOCX) [file pntd.0009083.s006.docx]

## **S2 Text: Publication bias**

For publication bias, we used Egger’s method (1) with significance $p-value <0\cdot05$ depicting publication bias. For *S. haematobium****,*** the Egger’s test p-value = 0.0177 and thus shows some level of publication bias. For *S. mansoni*, the Egger’s test p-value = 0.1554, which rules out significant publication bias.

**References**

1. Egger M, Smith GD, Schneider M, Minder C. Bias in meta-analysis detected by a simple, graphical test. Bmj. 1997;315(7109):629-34.
