# Supplementary material for: Gender-related differences in prevalence, intensity and associated risk factors of Schistosoma infections in Africa: A systematic review and meta-analysis
Source: PLoS Negl Trop Dis. 2021 Nov 17;15(11):e0009083. doi: 10.1371/journal.pntd.0009083 (PMC8635327; doi:10.1371/journal.pntd.0009083)
Supplement: S3 Text — (DOCX) [file pntd.0009083.s007.docx]

**S3 Text: Univariate meta-regression analysis**

We carried out a meta-regression analysis to test if age (lower and upper age limit), baseline prevalence and sample size are associated with the observed $M:F$ prevalence of infection ratios for each Schistosoma species separately. Association is determined via the Omnibus test (Q-test) where a p-value < 0.05 represents a significant association (1).

**References**

1. Hedges LV, Pigott TD. The power of statistical tests for moderators in meta-analysis. Psychological methods. 2004;9(4):426.
