# Supplementary material for: Gender-related differences in prevalence, intensity and associated risk factors of Schistosoma infections in Africa: A systematic review and meta-analysis
Source: PLoS Negl Trop Dis. 2021 Nov 17;15(11):e0009083. doi: 10.1371/journal.pntd.0009083 (PMC8635327; doi:10.1371/journal.pntd.0009083)
Supplement: S1 Fig — The funnel graph shows the effect measure (M:F prevalence ratio) and standard error (S.E.) for each study. (DOCX) [file pntd.0009083.s008.docx]

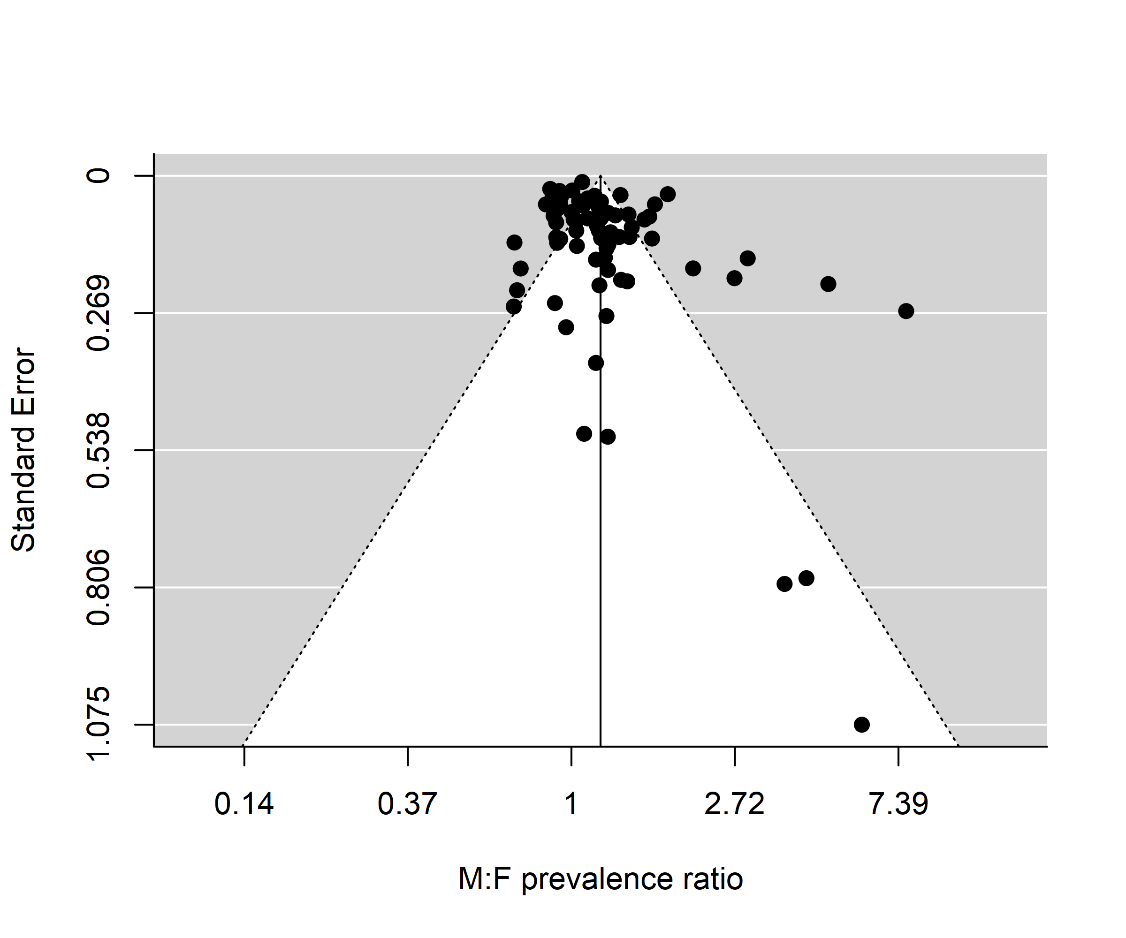


**S1 Fig** *:* Funnel plot for assessing publication bias in meta-analysis of 71 epidemiological studies in Africa of *S. haematobium*. The funnel graph shows the effect measure ($M:F$ prevalence ratio) and standard error (S.E.) for each study.
