# Supplementary material for: Gender-related differences in prevalence, intensity and associated risk factors of Schistosoma infections in Africa: A systematic review and meta-analysis
Source: PLoS Negl Trop Dis. 2021 Nov 17;15(11):e0009083. doi: 10.1371/journal.pntd.0009083 (PMC8635327; doi:10.1371/journal.pntd.0009083)
Supplement: S3 Fig — Forest plots showing the M:F prevalence ratios and 95% CI for S. haematobium according to sample size; a) Studies with sample size less than 2251(mean sample size of included studies), pooled M:F prevalence ratio is 1.17 (95% CI 1.08−1.27), I2 = 93.15%, and b) studies with sample size greater than 2251; M:F prevalence of infection ratio is 1.31 (95% CI 1.08−1.59), I2 = 98%. (DOCX) [file pntd.0009083.s010.docx]

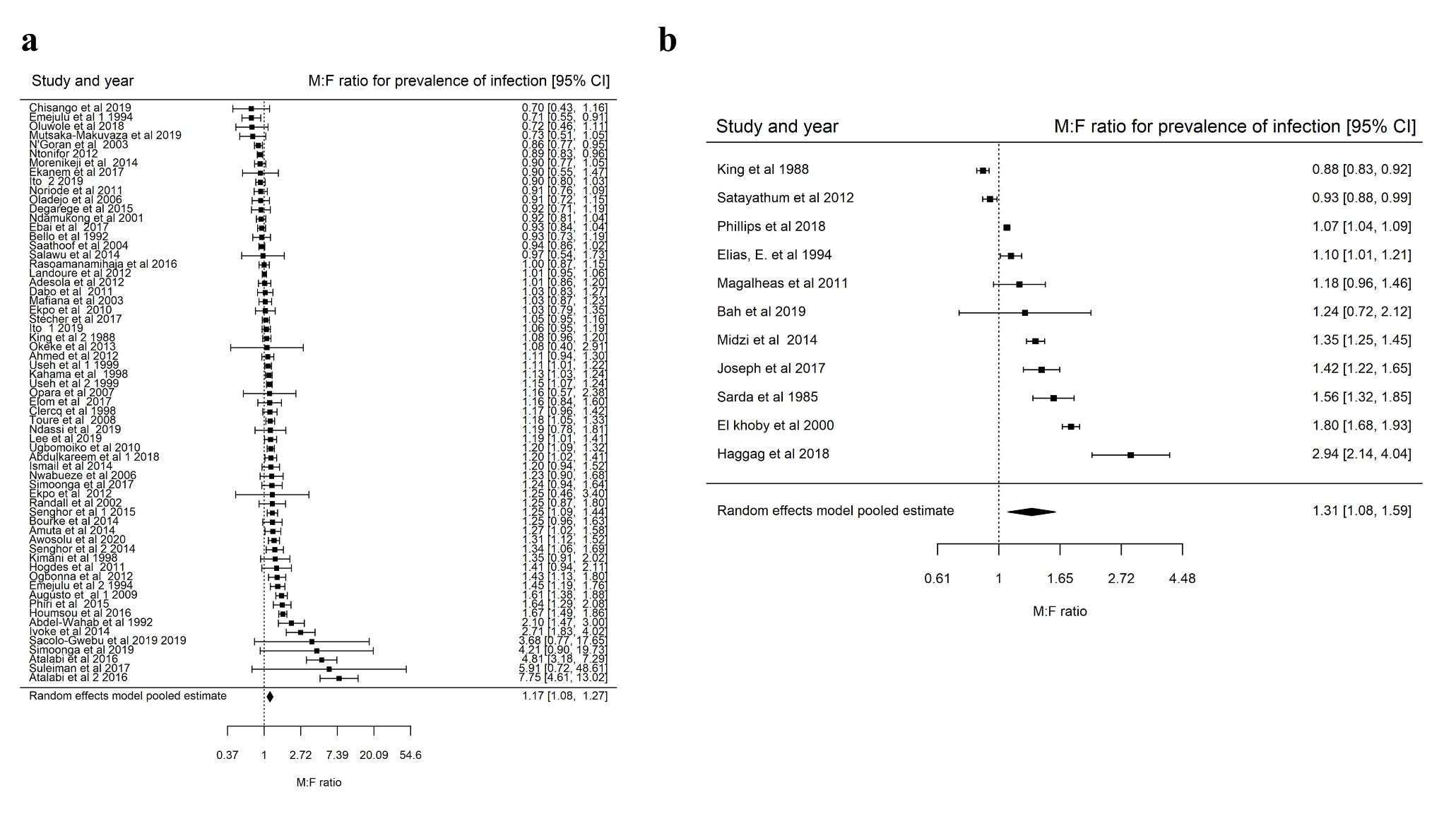


**S3 Fig** : Forest plots showing the $M:F$ prevalence ratios and 95% CI for *S. haematobium* according to sample size; a) Studies with sample size less than 2251(mean sample size of included studies), pooled $M:F$ prevalence ratio is $1.17 \left( 95\% CI 1.08-1.27 \right), I^{2}=93.15\%$, and b) studies with sample size greater than 2251; $M:F$ prevalence of infection ratio is $1.31 \left( 95\% CI 1.08-1.59 \right), I^{2}=98\%$.
