# Supplementary material for: Gender-related differences in prevalence, intensity and associated risk factors of Schistosoma infections in Africa: A systematic review and meta-analysis
Source: PLoS Negl Trop Dis. 2021 Nov 17;15(11):e0009083. doi: 10.1371/journal.pntd.0009083 (PMC8635327; doi:10.1371/journal.pntd.0009083)
Supplement: S5 Fig — Forest plots showing the M:F prevalence ratios and 95% CI for S. haematobium according to baseline prevalence; a) Studies with baseline prevalence greater than 50% pooled M:F prevalence ratio is 1.09 (95% CI 1.03−1.15), I2 = 91.40%, and b) studies with baseline prevalence less than 50%; M:F prevalence of infection ratio is 1.34 (95% CI 1.16−1.55), I2 = 94.42%. Analyses includes studies that report the number of individuals who were screened for S. haematobium infection and the fraction who tested positive distributed by sex regardless of the reported significance of the difference in M:F prevalence ratios. (DOCX) [file pntd.0009083.s012.docx]

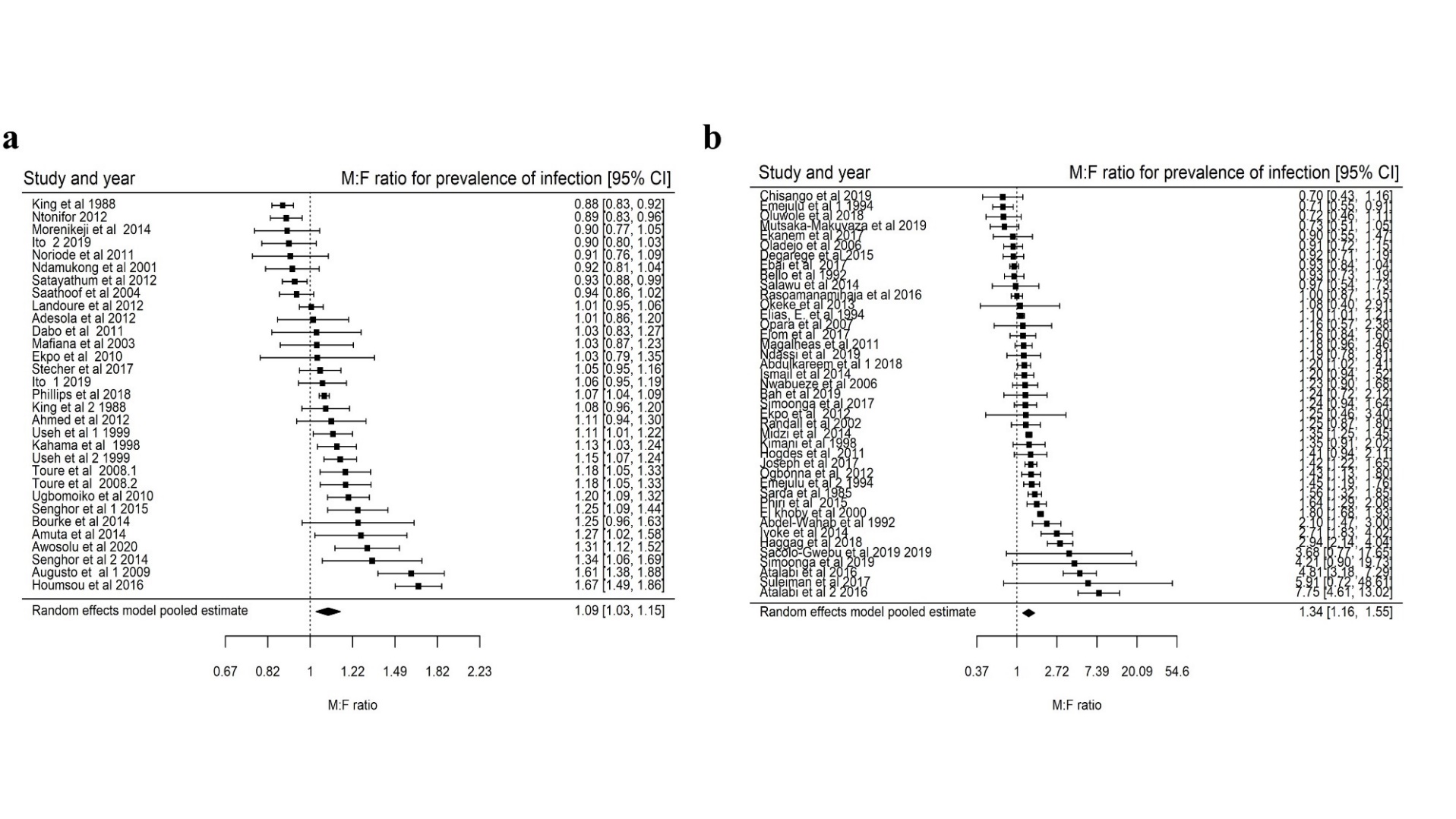


**S5 Fig** : Forest plots showing the $M:F$ prevalence ratios and 95% CI for *S. haematobium* according to baseline prevalence; a) Studies with baseline prevalence greater than 50% pooled $M:F$ prevalence ratio is $1.09 \left( 95\% CI 1.03-1.15 \right), I^{2}=91.40\%$, and b) studies with baseline prevalence less than 50%; $M:F$ prevalence of infection ratio is $1.34 \left( 95\% CI 1.16-1.55 \right), I^{2}=94.42\%$. Analyses includes studies that report the number of individuals who were screened for *S. haematobium* infection and the fraction who tested positive distributed by sex regardless of the reported significance of the difference in $M:F$ prevalence ratios.
