# Supplementary material for: Gender-related differences in prevalence, intensity and associated risk factors of Schistosoma infections in Africa: A systematic review and meta-analysis
Source: PLoS Negl Trop Dis. 2021 Nov 17;15(11):e0009083. doi: 10.1371/journal.pntd.0009083 (PMC8635327; doi:10.1371/journal.pntd.0009083)
Supplement: S6 Fig — Forest plots showing the M:F prevalence ratios and 95% CI for S. mansoni according to baseline prevalence; a) Studies with baseline prevalence greater than 50% pooled M:F prevalence ratio is 1.06 (95% CI 0.99−1.12), I2 = 90.38%, and b) studies with baseline prevalence less than 50%; M:F prevalence of infection ratio is 1.25 (95% CI 1.14−1.39), I2 = 97.84%. (DOCX) [file pntd.0009083.s013.docx]

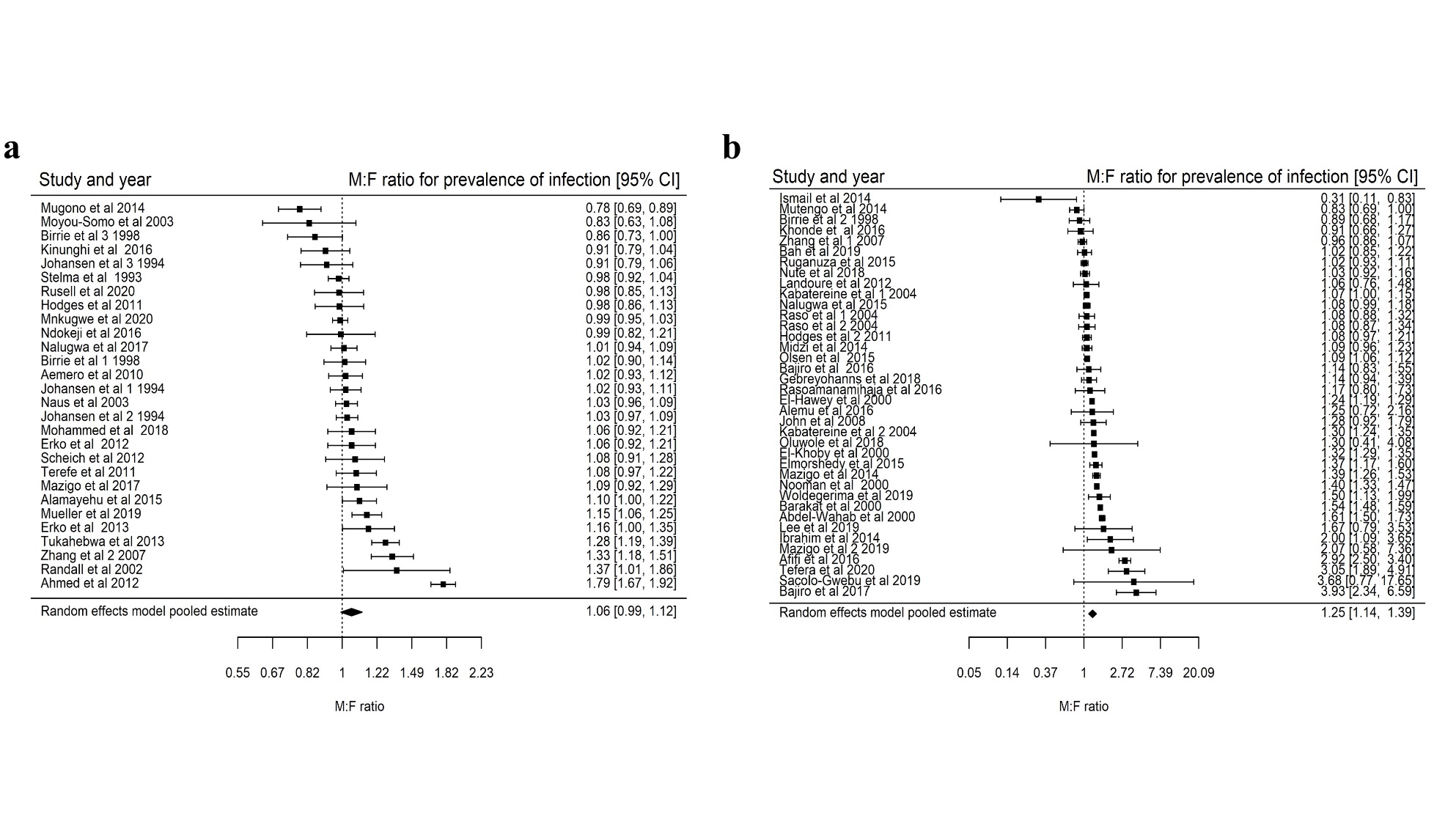


**S6 Fig:** Forest plots showing the $M:F$ prevalence ratios and 95% CI for *S. mansoni* according to baseline prevalence; a) Studies with baseline prevalence greater than 50% pooled $M:F$ prevalence ratio is $1.06 \left( 95\% CI 0.99-1.12 \right), I^{2}=90.38\%$, and b) studies with baseline prevalence less than 50%; $M:F$ prevalence of infection ratio is $1.25 \left( 95\% CI 1.14-1.39 \right), I^{2}=97.84\%$.
